# Glutamine Metabolism Underlies the Functional Similarity of T Cells between Nile Tilapia and Tetrapod

**DOI:** 10.1002/advs.202201164

**Published:** 2023-03-08

**Authors:** Kang Li, Xiumei Wei, Xinying Jiao, Wenhai Deng, Jiaqi Li, Wei Liang, Yu Zhang, Jialong Yang

**Affiliations:** ^1^ State Key Laboratory of Estuarine and Coastal Research School of Life Sciences East China Normal University Shanghai 200241 China; ^2^ Laboratory for Marine Biology and Biotechnology Qingdao National Laboratory for Marine Science and Technology Qingdao 266237 China; ^3^ School of Laboratory Medicine and Life Science Wenzhou Medical University Wenzhou Zhejiang 325035 China

**Keywords:** evolution, functional similarity, glutamine metabolism, T cells, tilapia

## Abstract

As the lowest organisms possessing T cells, fish are instrumental for understanding T cell evolution and immune defense in early vertebrates. This study established in Nile tilapia models suggests that T cells play a critical role in resisting *Edwardsiella piscicida* infection via cytotoxicity and are essential for IgM^+^ B cell response. CD3 and CD28 monoclonal antibody crosslinking reveals that full activation of tilapia T cells requires the first and secondary signals, while Ca^2+^–NFAT, MAPK/ERK, NF‐*κ*B, and mTORC1 pathways and IgM^+^ B cells collectively regulate T cell activation. Thus, despite the large evolutionary distance, tilapia and mammals such as mice and humans exhibit similar T cell functions. Furthermore, it is speculated that transcriptional networks and metabolic reprogramming, especially c‐Myc‐mediated glutamine metabolism triggered by mTORC1 and MAPK/ERK pathways, underlie the functional similarity of T cells between tilapia and mammals. Notably, tilapia, frogs, chickens, and mice utilize the same mechanisms to facilitate glutaminolysis‐regulated T cell responses, and restoration of the glutaminolysis pathway using tilapia components rescues the immunodeficiency of human Jurkat T cells. Thus, this study provides a comprehensive picture of T cell immunity in tilapia, sheds novel perspectives for understanding T cell evolution, and offers potential avenues for intervening in human immunodeficiency.

## Graphical abstract

Fish represent the lowest extant animals to possess T cells. Despite large evolutionary distance, tilapia share similar T cell function with mammals. Metabolic reprogramming, especially c‐Myc‐mediated glutamine metabolism, which is conserved across tilapia, frogs, chickens, and mice, underlies functional similarity of T cells between tilapia and tetrapods. Rebuilding glutaminolysis pathways using tilapia components rescues the immunodeficiency of human T cells.

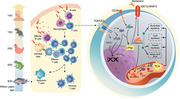

## 1 Introduction

T cells are specialized lymphocytes expressing uniquely rearranged T cell receptor (TCR) and represent a key component of the adaptive immune system. T cells are further classified into cytotoxic, helper, and regulatory subsets, which play key roles in pathogen resistance, tumor surveillance, and activation of other immune cells.^[^ ^1^, ^2^ ^]^ Given the prominent immunological properties, T cells have been increasingly used for immunotherapy in the last decade, for example, immune checkpoint blockade, adoptive cell therapy, and cancer vaccines.^[^ ^3^, ^4^, ^5^ ^]^ T cell function requires rewiring of distinct molecular machinery in response to physiologically relevant cues, and a fine‐tuned activation of T cells is central to the maintenance of immune homeostasis, otherwise autoimmune diseases and a loss of immune self‐tolerance may occur.^[^ ^6^ ^]^

T cell response is manipulated by networks comprising first and secondary signals, cytokines, and transcription factors. Recently, emerging evidence highlights the critical role of metabolic programs in T cell activation, proliferation, and differentiation. Shortly after activation, transporters ASCT2 and SNAT2 are upregulated in T cells, enhancing glutamine uptake;^[^ ^7^, ^8^ ^]^ glutamine is converted to glutamate in the mitochondria by glutaminase (GLS) and subsequently converted to *α*‐ketoglutarate (*α*‐KG) by glutamate dehydrogenase (GLUD).^[^ ^9^ ^]^ Such glutaminolysis promotes T cell proliferation and function by supplying the key intermediates for nucleotide, protein, and lipid biosynthesis^[^ ^10^, ^11^ ^]^ and reinforces T cell response through epigenetic remodeling or maintenance of redox balance.^[^ ^12^, ^13^ ^]^ Dysregulation of glutamine metabolism profoundly compromises T cell fate and function. For example, glutamine deprivation inhibits T cell proliferation and cytokine production;^[^ ^14^ ^]^ ASCT2 disruption decreases glutamine import and suppresses Th1 and Th17 cell differentiation;^[^ ^8^ ^]^ and GLS1 deficiency impairs Th17 cell polarization by limiting the supply of *α*‐KG.^[^ ^15^ ^]^ Moreover, the conventional immune pathways and transcription factors that regulate T cell proliferation and differentiation, such as TCR, mTORC1, MAPK/ERK, HIF‐1*α*, and c‐Myc, also actively participate in glutamine metabolism by targeting ASCT2, GLS, or GLUD,^[^ ^7^, ^8^, ^10^, ^16^ ^]^ thus making the glutamine metabolic pathway a central hub and key checkpoint for T cell immunity.

T cells are not exclusive to mammals; the common ancestor of jawed vertebrates had already developed primitive T cells,^[^ ^17^ ^]^ and fish represent the lowest extant organisms to possess T cells.^[^ ^18^ ^]^ As that in mammals, RAG, MHC, and TCR lay the molecular foundation of fish T cells,^[^ ^19^ ^]^ and the pan T cells has been identified in many teleost species using CD3, LCK, and ZAP70 antibodies.^[^ ^20^, ^21^, ^22^ ^]^ After having undergone maturation in the thymus,^[^ ^23^, ^24^ ^]^ T cells circulate in the peripheral blood, spleen, and head kidney and may also reside in the intestine, gill, skin, and fin.^[^ ^25^ ^]^ In sea bass and large yellow croaker, T cells are capable to be activated by the mitogen phytohemagglutinin (PHA),^[^ ^20^, ^26^ ^]^ whereas T cells in olive flounder and tilapia respectively involve in the immune response against intracellular and extracellular pathogens.^[^ ^21^, ^22^ ^]^ Although anglerfish and Atlantic cod don't have the MHCII/CD4 axis,^[^ ^27^ ^]^ most teleosts follow the configuration principle of CD8^+^ cytotoxic and CD4^+^ helper T cells. Teleost CD8^+^ T cell response can be induced by viral infection or mycolic acid stimulation.^[^ ^28^, ^29^ ^]^ Activated CD8*α* ^+^ lymphocytes in rainbow trout show induced expression of perforin, granulysin and IFN‐*γ*,^[^ ^30^ ^]^ but those in olive flounder upregulate FasL.^[^ ^31^ ^]^ CD8*α* ^+^ lymphocytes in ginbuna crucian carp exhibit cytotoxicity to allogeneic cells,^[^ ^32^ ^]^ and memory effects toward secondary infection.^[^ ^33^, ^34^ ^]^ In most teleost species, CD4^+^ T cells encode two CD4 receptors—CD4‐1 and CD4‐2, of which CD4‐1 is considered homologous to mammalian CD4.^[^ ^35^ ^]^ The facts that Japanese flounder possesses CD4‐1^+^T‐bet^+^ lymphocytes,^[^ ^36^ ^]^ tilapia T cells secrete IL‐17A to resist extracellular bacterial infection,^[^ ^37^ ^]^ and CD4‐1^+^ T cells from HGG‐immunized zebrafish upregulate IFN‐*γ*, IL‐4, and IL‐17 expression,^[^ ^38^ ^]^ collectively indicate the potential existence and immunological function of Th‐like cells in these fishes. In addition, zebrafish CD40L promotes the thymus dependent antigen‐induced IgM^+^ B cell proliferation and IgM production, which are inhibited by cyclosporin A (CyA)‐mediated T cell suppression, suggesting that teleost T cells also regulate humoral immunity.^[^ ^39^ ^]^ Despite these findings, our understanding of T cell immunity in teleost is rudimentary compared with that in mouse, because current knowledge is based on studies performed in diverse fish species belonging to different genera, families, and even orders, and their inherent differences make it challenging to consolidate these findings to obtain a comprehensive landscape. Therefore, systematic studies in a single species are necessary for the comprehensive understanding of T cell immunity in fish.

The lack of knowledge about elaborate mechanism is another obstacle to comprehensively understand the T cell immunity in teleost. Compared with the extensive functional research, only a few detail mechanisms regarding T cell immunity have been identified in teleost species. For example, the *γ*c cytokines IL‐7, IL‐2, and IL‐15 collectively drive T cell development in zebrafish,^[^ ^40^ ^]^ while IL‐2‐mediated JAK/STAT5 signaling coordinates the MAPK and mTORC1 axes to control T cell expansion in large yellow croaker.^[^ ^41^ ^]^ Previously, we suggested that Ca^2+^–calcineurin axis triggers the nuclear translocation of NFAT, and NF‐*κ*B coupled with TCR and IL‐17 signals to ensure the proper T cell activation, proliferation, and anti‐infection immunity in tilapia.^[^ ^37^, ^42^ ^]^ Remarkably, we found that mTORC1‐mediated metabolic reprogramming and MAPK/ERK‐controlled glycolysis jointly promote T cell response of tilapia.^[^ ^43^, ^44^ ^]^ However, whether and how other metabolic programs regulate T cell immunity in teleost remain unknown.

In the present study, using models established in Nile tilapia, we examined the primary and secondary immune response of T cells, investigated their proliferation, apoptosis, and cytotoxicity, and revealed their role in helping humoral response. Moreover, the signaling pathways underpinning T cell activation, and the indispensable assistance of B cells to this process were elucidated. We also examined the transcriptional and metabolic profiles of tilapia T cells, and found that mTORC1 coordinates MAPK/ERK signaling to promote T cell immunity via c‐Myc‐mediated glutamine metabolism. Intriguingly, T cell response in tilapia, frog, chicken, and mouse exhibited a conserved dependency on glutamine metabolism, and restoration of the glutaminolysis axis using tilapia components rescued the immunodeficiency of human T cells. These findings thus provide a comprehensive T cell landscape regarding immunological process, function, and mechanism in a single fish species, and suggest that the conserved signaling pathways, transcriptional networks, and metabolic programs, especially glutamine metabolism, support the functional similarity of T cells between tilapia and tetrapod. This study would provide novel insights for understanding T cell evolution and intervening immunodeficiency in human using a synthetic immunological approach.

## 2 Results

### 2.1 Nile Tilapia Requires T Cells to Resist Bacterial Infection

**Figure 1:**
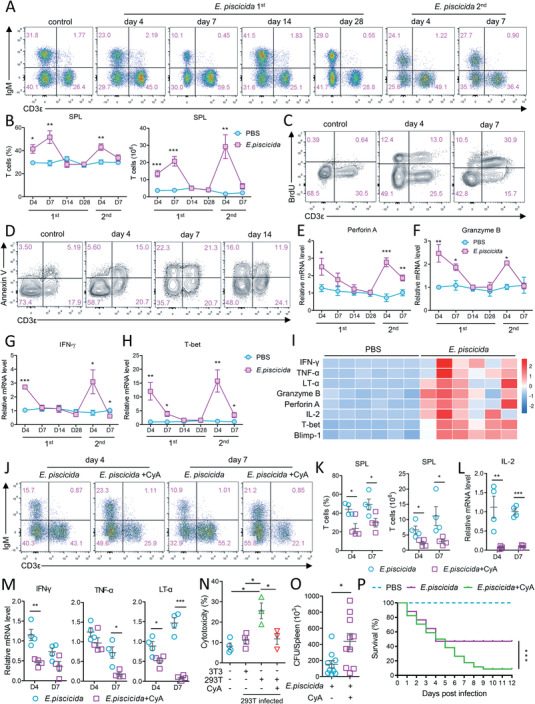
The processes and functional properties of T cell response in tilapia. Spleen leukocytes of tilapia that i.p. injected with *E. piscicida* on day 0 and day 30 were isolated on indicated days. A) Flow cytometry shown frequencies of T cells and IgM^+^ B cells in gated lymphocyte population. B) Frequencies (left) and absolute numbers (right) of T cells, *n* = 4–6. C) Tilapia was i.p. injected with 0.75 mg BrdU one day before scarification and BrdU incorporation of gated spleen lymphocytes was examined. D) Annexin V and CD3*ε* staining of spleen lymphocytes. E–H) mRNA levels of indicated molecules in spleen leukocytes, *n* = 4–5. I) Heatmap shown mRNA levels of indicated molecules in spleen T cells sorted from control or *E. piscicida*‐infected tilapia on 5 DPI, *n* = 6. Tilapia infected with *E. piscicida* or not were i.p. injected with 10 mg kg^−1^ cyclosporine A (CyA) on day 1, 3, 5, and 6. J) Representative dot plots shown frequencies of T cells and IgM^+^ B cells in gated spleen lymphocytes. K) Frequencies (left) and absolute numbers (right) of spleen T cells. L,M) Relative mRNA levels of indicated molecules in spleen leukocytes. N) Healthy tilapia were injected with 293T cells on day 1 and 3, and treated with 10 mg kg^−1^ CyA on day 1, 3, and 5. Spleen T cells were sorted on day 6 and incubated with 293T or NIH3T3 cells for 8 h, and LDH release was detected. O) Colonization of bacterial loads in spleen on 5 DPI. P) Kaplan–Meyer survival plot shown the survival percentage with or without CyA treatment post‐infection, *n* = 35. These experiments were repeated for three independent times. *: *p* < 0.05, **: *p* < 0.01, ***: *p* < 0.001, determined by a two‐tailed Student's *t*‐test.

To study T cell function, mAbs against Nile tilapia CD3*ε* (Figure S1, Supporting Information) and IgM (Figure S2A–H, Supporting Information) were developed, using which the T cell and B cell lineages in tilapia were identified (Figure S2I, Supporting Information). To investigate the T cell response in vivo, an infection model using intracellular bacteria *Edwardsiella piscicida* was established in tilapia (Figure S3A, Supporting Information). Compared with the uninfected controls (Figure S3B, Supporting Information), the number of spleen lymphocytes (Figure S3C, Supporting Information), and percentage and number of T cells (**Figure** **1**A,B) began to increase at 4 days post infection (DPI), and the expansion reached a peak at 7 DPI before T cell population contracted to the original level 2 weeks after infection (Figure 1A,B). The surviving animals were then infected again at 30 DPI. At 4 days after the secondary infection, the number of T cells was already more than the highest level observed at 7 days after the first infection (Figure 1A,B), indicating that the secondary infection triggered a more rapid T cell response. Furthermore, we found that the expansion of the T cell lineage resulted from cellular proliferation, as evidenced by the robust BrdU incorporation in these cells (Figure 1C); the continuous apoptosis accounted for the substantial reduction of T cells after the primary response (Figure 1D). As another arm of adaptive immunity, humoral response initiates later than cellular one; however, the rapid expansion during secondary infection was also applicable to IgM^+^ B cells (Figure 1A; Figure S3D, Supporting Information).

Next, we addressed the detail function of tilapia T cells. Owing to the high proportion (>90%) of lymphocytes (Figure S3E, Supporting Information) in our isolated spleen leukocytes, the latter were regarded as spleen lymphocytes and used for subsequent experiments. The mRNA levels of CD8*α* and Blimp‐1, which promote CD8^+^ effector T cell differentiation,^[^ ^45^ ^]^ were markedly upregulated in spleen lymphocytes during the primary and secondary immune response (Figure S3F,G, Supporting Information). Moreover, inducible expression of the cytotoxic genes Perforin A, Granzyme B, IFN‐*γ*, TNF‐*α*, and LT‐*α* was also observed (Figure 1E–G; Figure S3H,I, Supporting Information). In addition, *E. piscicida* infection significantly induced the mRNA expression of co‐receptor CD4‐1 (Figure S3J, Supporting Information) and transcription factor T‐bet in spleen lymphocytes (Figure 1H), but hardly or slightly for GATA‐3 or ROR*α* (Figure S3K,L, Supporting Information). Moreover, upregulation of the above‐mentioned genes was further confirmed in sorted CD3^+^ T cells at 5 DPI (Figure 1I; Figure S3M, Supporting Information). To further assess the T cell function, tilapia was treated with the T cell‐specific inhibitor CyA^[^ ^39^, ^46^ ^]^ during bacterial infection. The proportion and absolute number of T cells in CyA‐treated tilapia were markedly lower than those in untreated counterparts (Figure 1J,K), and the BrdU incorporation in CD3^+^ T cells was impaired in CyA‐treated tilapia (Figure S3N, Supporting Information). Consistent with these observations, CyA treatment severely impaired the inducible expression of IL‐2 (Figure 1L) and the cytotoxic genes IFN‐*γ*, TNF‐*α*, and LT‐*α* in spleen lymphocytes (Figure 1M), and dampened the cytotoxicity of CD3^+^ T cells (Figure 1N). The deficiency and reduced cytotoxicity of T cells made it challenging to manage the infection (Figure 1O) and rendered the tilapia more vulnerable to pathogen infection (Figure 1P). Overall, these results reveal the immunological processes and indispensable roles of T cells during the anti‐bacterial response in tilapia.

### 2.2 T Cells are Indispensable for Proper B Cell Response in Tilapia

**Figure 2:**
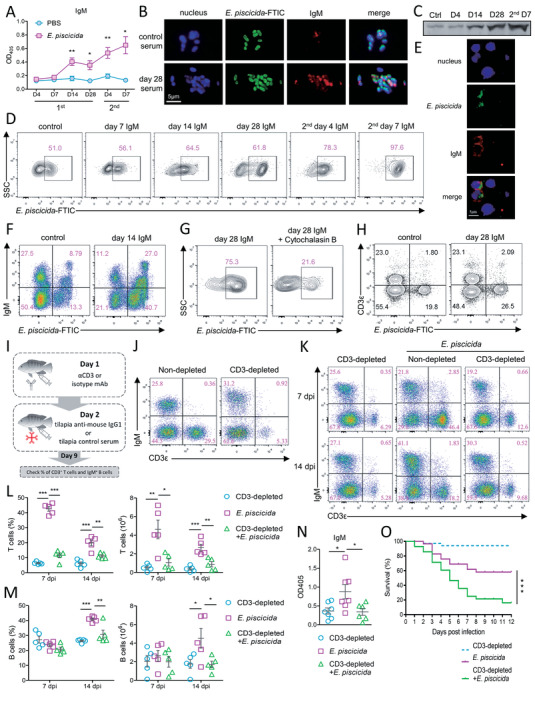
Tilapia T cells facilitate IgM^+^ B cell‐mediated humoral response. A) Relative amounts of serum IgM in *E. piscicida*‐infected or uninfected tilapia, *n* = 3–5. *E. piscicida* was incubated with serum from *E. piscicida*‐infected or uninfected tilapia for 2 h, and B) immunofluorescence and C) western blot assay shown the interaction of *E. piscicida* with serum IgM. After incubated with the indicated serum IgM, FITC‐labelled *E. piscicida* was incubated with head kidney leukocytes in vitro with or without 0.08 mg mL^−1^ Cytochalasin B for 6 h. Flow cytometry and immunofluorescence shown the phagocytosis of head kidney D) myeloid cells, E–G) IgM^+^ B cells, or H) T cells to *E. piscicida* that coated with serum IgM from infected or uninfected tilapia. I) Strategy for T cells depletion. J) Frequencies of T cells and IgM^+^ B cells in spleen of CD3*ε*‐depleted or non‐depleted tilapia on 9‐day post depletion. CD3*ε*‐depleted or non‐depleted tilapia was infected with *E. piscicida* or not. K) Representative dot plots, L) frequencies (left) and absolute numbers (right) of T cells, M) IgM^+^ B cells in spleen on indicated day post infection, N) relative amounts of serum IgM on 14 DPI, and O) the survival percentage of tilapia (*n* = 25–26) were shown. These experiments were repeated for three independent times. *: *p* < 0.05, **: *p* < 0.01, ***: *p* < 0.001, determined by a two‐tailed Student's *t*‐test.

In addition to exerting the cellular response, T cells help B cells to orchestrate the humoral immunity. Pathogen‐induced IgM production has been reported in several other teleosts.^[^ ^47^, ^48^ ^]^ In tilapia, the serum IgM concentration began to increase at 14 DPI and was further elevated in response to secondary infection (**Figure** **2**A), which is consistent with the IgM^+^ B cell response described above. It has been reported that fish myeloid cells and IgM^+^ B cells phagocytize microbes.^[^ ^49^, ^50^ ^]^ In the present study, serum IgM from *E. piscicida*‐infected tilapia, especially after secondary infection, coated the pathogen (Figure 2B,C) and enhanced the phagocytosis by myeloid cells (Figure 2D) and IgM^+^ B cells (Figure 2E,F). Meanwhile, the enhanced phagocytosis was impaired when the cells were treated with cytochalasin B (Figure 2G), suggesting the specific opsonic function of tilapia IgM. In contrast, tilapia T cells could not phagocytize *E. piscicida* or IgM‐coated *E. piscicida* (Figure 2H). To determine whether the humoral immune response mounted by IgM and IgM^+^ B cells is dependent on T cells, we employed a model in which we were able to selectively deplete the T cell lineage in adult animals using anti‐tilapia CD3*ε* mAb in combination with tilapia anti‐mouse IgG1 serum (Figure 2I). On day 9 after antibody administration, more than 80% of CD3^+^ T cells were depleted compared with the isotype control antibody group (Figure 2J), and the effect lasted for another 3 weeks. The T cell‐depleted or nondepleted tilapia was then infected with *E. piscicida*. At 7 DPI, a robust expansion of T cells was induced in nondepleted tilapia but not in T cell‐depleted individuals (Figure 2K,L). At 14 DPI, IgM^+^ B cells in nondepleted fish had already undergone expansion (Figure 2K,M), but the lack of T cells in T cell‐depleted tilapia (Figure 2K,L) resulted in a defect of IgM^+^ B cell expansion (Figure 2K,M) and an impaired IgM production (Figure 2N). The deficiency of T cells, IgM^+^ B cells, and IgM collectively caused high mortality in tilapia during the bacterial infection (Figure 2O). Thus, these findings suggest that T cells are critical for proper B cell expansion and antibody production in tilapia.

### 2.3 Tilapia Utilizes Classical Strategies to Modulate T Cell Activation

**Figure 3:**
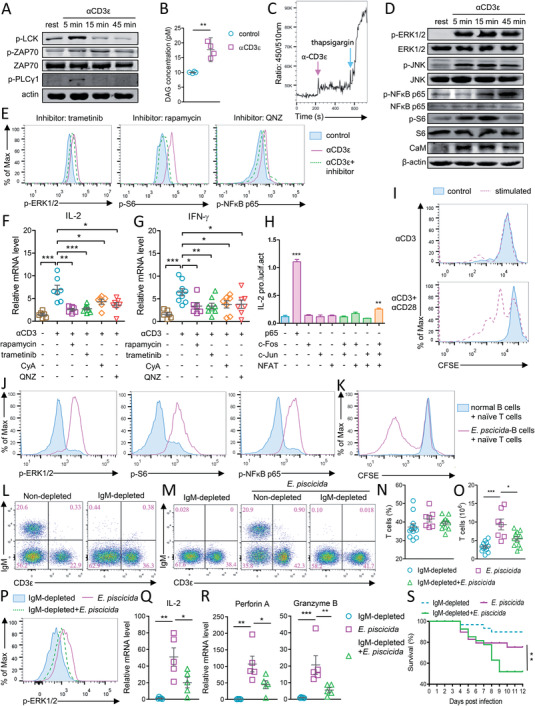
Tilapia utilize conserved signals and B cell help to activate T cells. Spleen leukocytes were stimulated with 2 µg mL^−1^ CD3*ε* mAb in the presence or absence of indicated inhibitor. A,D) Expressions of the indicated molecules were examined by western blot. B) Concentration of DAG at 45 min after stimulation. C) Ca^2+^ influx examined by flow cytometry after CD3*ε* mAb or 2 µg mL^−1^ thapsigargin stimulation. E) Phosphorylation of indicated molecules in T cells at 6 h after stimulation. F,G) mRNA levels of the indicated molecules were determined at 12 h after stimulation. H) HEK 293T cells were co‐transfected with IL‐2 promoter and indicated transcription factors, and IL‐2 promoter activities were examined by dual‐luciferase assay at 48 h post transfection, *n* = 4. I) CFSE‐labelled spleen leukocytes were stimulated with 2 µg mL^−1^ CD3*ε* plus CD28 mAbs, and T cell proliferation was examined by flow cytometry at 72 h. Spleen T cells and IgM^+^ B cells were sorted from one same tilapia individual. IgM^+^ B cells were incubated with inactivated *E. piscicida* for 6 h, before they were co‐cultured with T cells. J) Phosphorylation of indicated molecules in T cell population at 6 h. K) CFSE dilution assay shown the proliferation of T cell at 48 h. L) Frequencies of T cells and IgM^+^ B cells in spleen of IgM‐depleted or non‐depleted tilapia on 9‐day post depletion. IgM‐depleted or non‐depleted tilapia was infected with *E. piscicida* or not. M) Representative dot plots, N) frequencies, and O) absolute numbers of T cells in spleen on 7‐day post infection. P) Phosphorylation of ERK1/2 in T cells on 5‐day post infection. Q,R) mRNA levels of indicated molecules in sorted spleen T cells on 5‐day post infection. S) Kaplan–Meyer survival plot shown the survival percentage of tilapia, *n* = 25–26. These experiments were repeated for three independent times. *: *p* < 0.05, **: *p* < 0.01, ***: *p* < 0.001, determined by a two‐tailed Student's *t*‐test.

To ensure the proper immunological functions, T cells in mouse and human utilize multiple sophisticated signals to modulate their activation. Given that the CD3*ε* mAb crosslinking approach essential for investigating antigen‐induced T cell activation has been used exclusively in mammal studies, TCR signals remain unknown in teleost species. Here, we found that stimulation of spleen lymphocytes using CD3 mAb 2B2D7 enhanced the phosphorylation of LCK, ZAP‐70, and PLC*γ*1 (**Figure** **3**A), elevated diacyl glycerol concentration (Figure 3B), and triggered robust Ca^2+^ influx (Figure 3C), suggesting these early activation events occurring in tilapia T cells. In addition, CD3 mAb stimulation increased the phosphorylation of ERK1/2, JNK, NF‐*κ*B p65, and S6 and upregulated the expression of CaM in spleen lymphocytes (Figure 3D) and gated CD3^+^ T cells (Figure 3E), eventually leading to T cell activation, as evidenced by the elevated transcription of cytokine IL‐2 and IFN‐*γ* (Figure 3F,G). Since these results indicated a potential association between TCR downstream pathways and T cell activation, we next aimed to confirm this correlation. Overexpression of the transcription factor NF‐*κ*B p65 or the NFAT1‐AP1 (c‐Fos/c‐Jun) dimer markedly enhanced the promoter activity of IL‐2 (Figure 3H), suggesting the transcriptional regulation of tilapia IL‐2 by these pathways. Furthermore, blockade of MAPK/ERK, mTORC1, NF‐*κ*B, or the Ca^2+^‐NFAT axis with specific inhibitor (Figure 3E) markedly impaired the TCR stimulation‐induced upregulation of IL‐2 and IFN‐*γ* (Figure 3F,G), suggesting they are crucial for T cell activation in tilapia. In addition to TCR binding to antigen‐loaded MHC molecules, secondary signals are needed for T cell activation and response. To investigate whether full activation of tilapia T cells depends on secondary signal, we developed tilapia CD28 mAb (Figure S4A–D, Supporting Information). Stimulation with CD3*ε* plus CD28 mAbs, but not with only CD3*ε* mAb, induced robust proliferation of tilapia T cells in vitro (Figure 3I), suggesting that although the first signal is sufficient to initiate T cell activation, full activation and proliferation of tilapia T cells require secondary signal from CD28. Taken together, our findings suggest that the regulatory strategies used for T cell activation in tilapia are similar to those observed in mammals such as mouse and human.

### 2.4 Tilapia T Cells Require Assistance from B Cells for Optimal Activation and Function

T cell response relies on the assistance of other immune cells such as macrophages, dendritic cells, and B cells.^[^ ^1^, ^2^ ^]^ In mammals, B cells provide the first and secondary signals essential for T cell activation.^[^ ^51^ ^]^ In the present study, we examined the role of IgM^+^ B cells in the T cell response of tilapia. CD3^+^ T cells and IgM^+^ B cells were sorted from the same tilapia individual for experiments. Naïve T cells co‐cultured with *E. piscicida*‐loaded IgM^+^ B cells, but not with normal IgM^+^ B cells, exhibited enhanced phosphorylation of ERK1/2, S6, and NF‐*κ*B p65 (Figure 3J), and robust cell proliferation (Figure 3K), suggesting that the B cell lineage plays a crucial role in regulating T cell activation. Next, an IgM^+^ B cell depletion model was developed by injecting anti‐tilapia IgM mAb plus tilapia anti‐mouse IgG1 serum (Figure S4E, Supporting Information); at day 9 after antibody administration, almost all IgM^+^ B cells were selectively depleted compared with the isotype control group (Figure 3L). The B cell‐depleted and control tilapia were then infected with *E. piscicida*. At 7 DPI, although the percentage of T cells among lymphocytes was comparable between B cell‐depleted and nondepleted tilapia (Figure 3M,N), the numbers of lymphocytes and T cells were markedly reduced in IgM^+^ B cell‐depleted tilapia (Figure S4F,G, Supporting Information; Figure 3O). Furthermore, the increase in ERK1/2 phosphorylation and IL‐2 expression in gated or sorted T cells was markedly impaired in B cell‐depleted tilapia (Figure 3P,Q), indicating defective T cell activation in the absence of B cells. Moreover, the depletion of IgM^+^ B cells weakened the expression of cytotoxic genes in T cells (Figure 3R), consequently rendering the tilapia more vulnerable to bacterial infection (Figure 3S). Our findings collectively suggest that B cells are indispensable for optimal T cell activation and function in tilapia.

### 2.5 Metabolic Reprogramming is Employed to Fulfill the Requirements of T Cell Response in Tilapia

**Figure 4:**
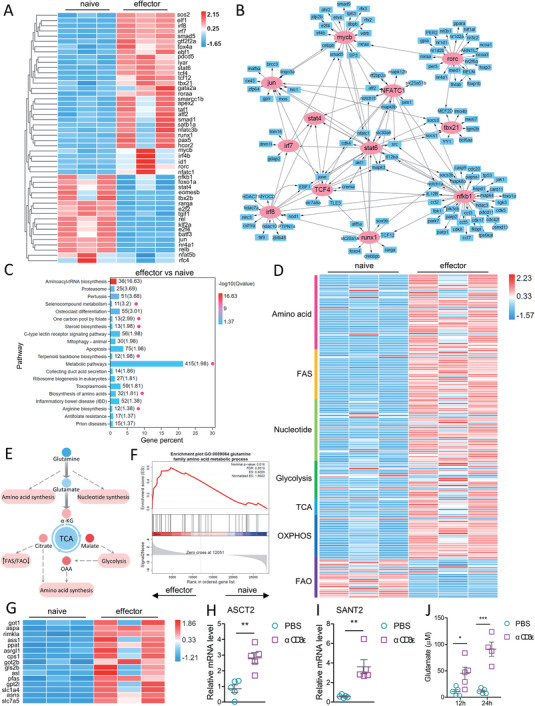
Transcription prolife and metabolic reprogramming of tilapia T cells. Spleen T cells were sorted from control or *E. piscicida*‐infected tilapia on 5 DPI, and subjected for RNA‐seq analyses. *n* = 3. A) Heatmap shown the different expression patterns of transcription factors between naïve and effector T cells, with *p* < 0.05 and |log2FC| > 1.5. B) Protein–protein interaction shown the predicted transcriptional networks guiding T cell response. C) KEGG pathway enrichment analysis of differentially expressed genes shown the Top‐20 pathways involved. FDR < 0.05 and |log2FC| >2. Scale bar represents z scores that were generated from −log10 (Qvalues). The red points represent the pathways related to metabolism. D) Heatmap shown the different expression patterns of metabolic genes of indicated pathways between naïve and effector T cells, with *p* < 0.05 and |log2FC| > 1.5. E) Schematic representation of metabolic network basing on glutaminolysis in mammalian T cells. F) Gene‐set enrichment analysis (GSEA) shown a significant enrichment of gene sets associated with glutamine metabolism. G) Heatmap shown the different expression patterns of glutamine metabolic genes between naïve and effector T cells, with *p* < 0.05 and |log2FC| > 1.5. Spleen leukocytes of tilapia were stimulated with 2 µg mL^−1^ CD3*ε* mAb. H,I) Relative mRNA levels of indicated molecules at 12 h post stimulation. J) Concentration of glutamate at 12 and 24 h post stimulation. Experiments in (H)–(J) were repeated for three independent times. *: *p* < 0.05, **: *p* < 0.01, ***: *p* < 0.001, determined by a two‐tailed Student's *t*‐test.

To elucidate the mechanism underpinning T cell response in tilapia, naïve and effector T cells from healthy or *E. piscicida*‐infected tilapia at 5 DPI were sorted for RNA sequencing (RNA‐seq) analysis (Table S1, Supporting Information). In total, 1935 differentially expressed genes (DEGs) were identified between effector and naïve T cells, and the GO enrichment analysis revealed that these DEGs were enriched in biological processes, cellular component, and molecular function terms (Figure S5A, Supporting Information). The heat map and potential interaction of transcription factors (**Figure** **4**A,B), suggests that tilapia T cells utilize transcriptional networks similar to those in mammals to regulate the primary immune response. However, at 5 DPI, the mRNA expression of certain transcription factors, including NF‐*κ*B, STAT4, Jun, and BATF, was downregulated in effector T cells (Figure 4A), which may be attributed to an inappropriate sampling time point. In addition, KEGG pathway enrichment analysis revealed that the top 20 pathways enriched by the DEGs involved five biological processes (Figure S5B, Supporting Information). Notably, more than 26% of the DEGs were associated with metabolic signaling (Figure 4C), and most of these metabolic genes were associated with lipid, amino acid, or carbohydrate metabolism (Figure S5C, Supporting Information). Furthermore, effector T cells showed marked upregulation of genes associated with amino acid metabolism, fatty acid synthesis, nucleotide synthesis, glycolysis, tricarboxylic acid (TCA) cycle, and Oxidative phosphorylation (OXPHOS) but downregulation of those involved in fatty acid oxidation (FAO) (Figure 4D), suggesting an obvious metabolic reprogramming occurred. This transition was also supported by the gene‐set enrichment analysis (GSEA) (Figure S5D, Supporting Information). Because amino acid, nucleotide, and fatty acid biosynthesis and the TCA cycle can be all metabolically traced to the same source—glutaminolysis (Figure 4E), we speculate that glutamine metabolism is an intrinsic pathway underpinning the T cell response in tilapia. GSEA showed a significant enrichment of gene sets associated with glutamine metabolism (Figure 4F); moreover, glutaminolysis‐related genes were markedly upregulated in effector T cells from *E. piscicida*‐infected tilapia (Figure 4G). In addition, in vitro activation of T cells induced the expression of glutamine transporters ASCT2 and SNAT2 (Figure 4H,I) and increased the transient accumulation of glutamate (Figure 4J), indicating the glutaminolysis associated with T cell activation. Thus, evolutionarily conserved transcriptional networks and metabolic reprogramming are utilized to fulfill the conditions required for the T cell response in tilapia, and glutamine metabolism may be central to this transition.

### 2.6 Glutamine and Its Metabolism are Pivotal for T Cell Response in Tilapia

**Figure 5:**
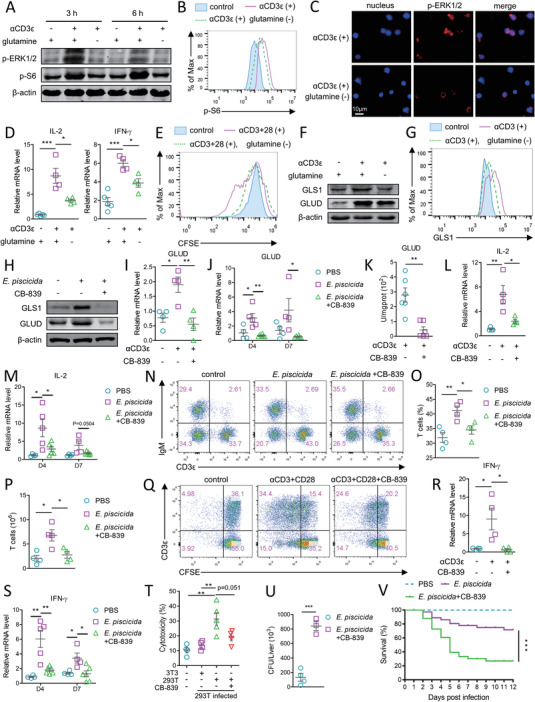
Glutamine metabolism ensures proper T cell function of tilapia. Spleen leukocytes of tilapia cultured in DMEM with or without glutamine were stimulated with A–D,F,G) 2 µg mL^−1^ CD3*ε* mAb or E) plus 2 µg mL^−1^ CD28 mAb. A) ERK1/2 and S6 phosphorylation at indicated time points. B) S6 phosphorylation in T cells were examined by flow cytometry at 6 h. C) ERK1/2 phosphorylation by immunofluorescence assay at 6 h. D) mRNA levels of indicated molecules at 12 h. E) CFSE‐labeled spleen leukocytes were stimulated with CD3*ε* and CD28 mAbs, and proliferation of T cells was examined at 72 h post stimulation. F) Protein levels of indicated molecules at 24 h. G) GLS1 expression in T cells were examined by flow cytometry at 24 h. H) Tilapia infected with *E. piscicida* were i.p. injected with 20 mg kg^−1^ CB‐839 on day 1, 2, and 3, and expression of indicated proteins in spleen leukocytes was examined on 4 DPI. I,K,L, Spleen leukocytes that stimulated with 2 µg mL^−1^ CD3*ε* mAb were treated with 10 nM CB‐839 or not for 24 h, and I,L) mRNA levels of indicated molecules and K) enzyme activity of GLUD were examined. J,M–P,S,U,V) Tilapia that infected with *E. piscicida* were injected with 20 mg kg^−1^ CB‐839 on day 1, 3, 5, and 6. J,M,S) The mRNA levels of indicated molecules in spleen leukocytes on 4 and 7 DPI, N,O) T cell percentages, and P) absolute numbers in spleen on 4 DPI, U) colonization of bacterial loads in live on 5 DPI, and V) the survival percentage of tilapia post‐infection (*n* = 30–32) were shown. Q) CFSE‐labeled spleen leukocytes were stimulated with 2 µg mL^−1^ CD3*ε* and CD28 mAbs with or without 10 nM CB‐839, and T cell proliferation was examined at 72 h. R) Relative mRNA levels of IFN‐*γ* in spleen leukocytes that stimulated with 2 µg mL^−1^ CD3*ε* for 12 h with or without 10 nM CB‐839. T) Healthy tilapia were injected with 293T cells on day 1 and 3, and treated with 20 mg kg^−1^ CB‐839 on day 1, 3, and 5. Spleen T cells were sorted on day 6 and incubated with 293T or NIH3T3 cells for 8 h, and LDH release was detected. These experiments were repeated for three independent times. *: *p* < 0.05, **: *p* < 0.01, ***: *p* < 0.001, determined by a two‐tailed Student's *t*‐test.

Glutamine, the most abundant nutrient in the blood, underpins T cell fate and function in human and mouse.^[^ ^10^, ^13^ ^]^ Considering that the habitat and dietary structure of tilapia differ from those of mammals, the contribution of glutamine and its metabolism to T cell immunity in tilapia needs further investigation. Since effector T cells of tilapia prefer glutamine metabolism, we first assessed the glutamine dependency of T cell immunity. In the presence of glutamine, CD3*ε* mAb stimulation enhanced ERK1/2 and S6 phosphorylation in spleen lymphocytes, whereas these activation events were markedly hindered upon glutamine deprivation (**Figure** **5**A). The reduced phosphorylation in the absence of glutamine was also confirmed in spleen T cells by flow cytometry (Figure 5B), and at the cellular level by immunofluorescence microscopy (Figure 5C; Figure S6A, Supporting Information). Notably, upon TCR stimulation, glutamine deprivation impaired the upregulation of IL‐2 and IFN‐*γ* in spleen lymphocytes (Figure 5D), and diminished T cell proliferation (Figure 5E). These observations collectively indicate an indispensable role of glutamine in maintaining the proper immunological function of T cells in tilapia.

Next, we investigated the effects of glutamine metabolism on T cell response in tilapia. An in‐depth exploration of the Nile tilapia genome identified all components of the glutaminolysis pathway, including SNAT2, ASCT2, GLS1, GLUD, and the aminotransferase GPT and GOT (Figure S6B, Supporting Information). The functional domains, primary structure, and tertiary structure of these components, especially the two key enzymes GLS1 and GLUD, were found to be highly conserved during evolution (Figure S6C–J, Supporting Information). Moreover, T cell activation caused an inducible expression of GLS1 and GLUD in spleen lymphocytes or T cells, and the upregulation of these molecules was impaired under glutamine deprivation (Figure 5F,G), which indicates a potential association between T cell immunity and glutamine metabolism in tilapia. To confirm the correlation, GLS1 inhibitor CB‐839 was used to block glutaminolysis. In activated spleen lymphocytes, CB‐839 treatment severely impaired the increase of GLS1 and GLUD (Figure 5H–J) and weakened GLUD activity (Figure 5K), indicating a defective glutaminolysis. Furthermore, inhibition of glutaminolysis impaired the inducible expression of IL‐2 in spleen lymphocytes upon CD3 mAb stimulation (Figure 5L) or *E. piscicida* infection (Figure 5M), hindered T cell expansion in vivo (Figure 5N–P) and in vitro (Figure 5Q), reduced the ability of activated lymphocytes to produce IFN‐*γ* (Figure 5R,S), and dampened the cytotoxicity of sorted T cells (Figure 5T). These harmful effects collectively resulted in a marked defect in elimination of infection (Figure 5U) and rendered the tilapia more susceptible to the pathogen (Figure 5V). Overall, these results suggest that, in tilapia, glutamine metabolism is not merely favored by effector T cells; it tightly regulates T cell function.

### 2.7 c‐Myc‐Regulated Glutamine Metabolism Ensures the T Cell Response of Tilapia

**Figure 6:**
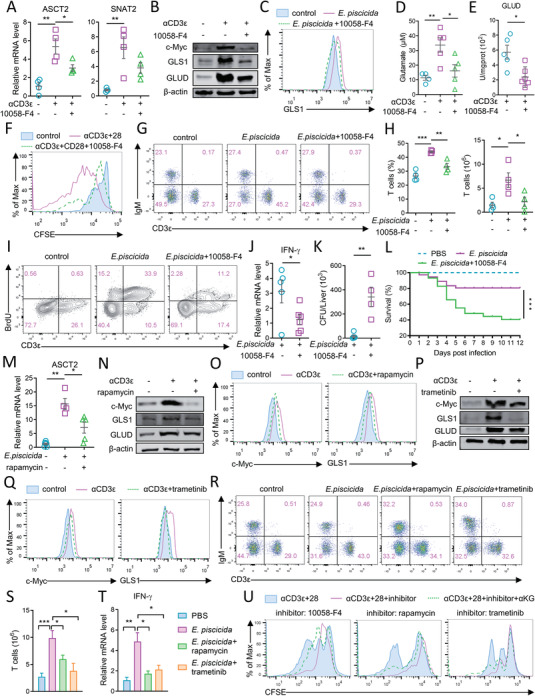
mTORC1 coordinates MAPK/ERK axis to promote T cell response of tilapia via c‐Myc‐regulated glutaminolysis. Spleen leukocytes that stimulated with 2 µg mL^−1^ CD3*ε* mAb or plus 2 µg mL^−1^ CD28 mAb were treated with or without 5 nM 10058‐F4. A) Relative mRNA or B) protein levels of indicated molecules, D) abundances of glutamate, and E) enzyme activity of GLUD were examined at 12 h. F) Proliferation of CFSE‐labeled T cells were examined at 72 h. Healthy or *E. piscicida*‐infected tilapia were i.p. injected with 10 mg kg^−1^ 10058‐F4 on day 1, 3, 5 and 6. C) GLS1 expression in spleen T cells on 4 DPI, G,H) frequencies and absolute numbers of spleen T cells on 7 DPI, I) BrdU incorporation of spleen T cells on 5 DPI, J) relative mRNA levels of IFN‐*γ* in spleen leukocytes on 4 DPI, K) colonization of bacterial loads in spleen on 5 DPI, and L) survival percentage of tilapia post infection (*n* = 25–30) were examined. M) Healthy or *E. piscicida*‐infected tilapia was i.p. injected with 1 mg kg^−1^ rapamycin on day 1, 2, and 3, and relative mRNA level of ASCT2 in spleen leukocytes was examined on 4 DPI. Tilapia individuals were i.p. injected with 1 mg kg^−1^ rapamycin or 0.1 mg kg^−1^ trametinib for consecutive 3 days, before the spleen leukocytes were isolated and stimulated with 2 µg mL^−1^ CD3*ε* mAb for 12 h. N,P) The expression of indicated molecules in spleen leukocytes or O,Q) T cells were examined. Healthy or *E. piscicida*‐infected tilapia was i.p. injected with 1 mg kg^−1^ rapamycin or 0.1 mg kg^−1^ trametinib for consecutive 3 days, and spleen leukocytes were isolated on 4 DPI. R) Flow cytometry and S) bar figures shown frequencies and absolute numbers of spleen T cells, *n* = 4–9. T) Relative mRNA levels of IFN‐*γ* in spleen leukocytes on 4 DPI, *n* = 5–6. U) CFSE‐labeled spleen leukocytes were stimulated with 2 µg mL^−1^ CD3*ε* plus CD28 mAbs with or without indicated inhibitor and additional 1 mm *α*‐KG, and proliferation of T cells were examined at 72 h after stimulation. These experiments were repeated for three independent times. *: *p* < 0.05, **: *p* < 0.01, ***: *p* < 0.001, determined by a two‐tailed Student's *t*‐test.

We investigated the mechanism by which tilapia modulates glutamine metabolism and subsequent T cell response. Given that c‐Myc regulates glutamine metabolism at multiple levels,^[^ ^52^, ^53^ ^]^ and spleen lymphocytes and sorted T cells of tilapia showed inducible c‐Myc expression during anti‐bacterial response (Figure S7A,B, Supporting Information) or CD3 mAb‐induced T cell activation (Figure S7C, Supporting Information), we aimed to examine the corresponding effect of c‐Myc on glutaminolysis‐promoted T cell response in tilapia. In tilapia treated with the c‐Myc inhibitor 10058‐F4, the upregulation of c‐Myc expression in spleen lymphocytes during CD3 mAb stimulation (Figure S7C, Supporting Information) or bacterial infection (Figure S7D, Supporting Information) was markedly suppressed, which further impaired the upregulation of ASCT2, SNAT2, GLS1, and GLUD (**Figure** **6**A; Figure S7E–G, Supporting Information). Consistent with these transcriptional findings, GLS1 and GLUD protein levels were also reduced once c‐Myc was inhibited (Figure 6B; Figure S7H, Supporting Information). More precisely, 10058‐F4 treatment impaired c‐Myc and GLS1 expression in activated T cells (Figure S7I, Supporting Information; Figure 6C), which consequently reduced glutamate abundance (Figure 6D) and decreased GLUD activity upon T cell activation (Figure 6E). Since these observations demonstrate that c‐Myc regulates glutamine metabolism of tilapia T cells, we next aimed to assess the association between c‐Myc‐mediated glutaminolysis and T cell immunity. c‐Myc inhibition impaired T cell proliferation upon CD3 mAb stimulation (Figure 6F), and diminished the expansion of lymphocytes and T cells during *E. piscicida* infection, as indicated by the reduced cell percentage and number (Figure S7J, Supporting Information; Figure 6G,H) and decreased BrdU incorporation (Figure 6I). Moreover, c‐Myc blockade compromised the IFN‐*γ* expression and infection elimination (Figure 6J,K), eventually leading to a higher mortality (Figure 6L). Taken together, these findings suggest that c‐Myc promotes glutaminolysis to ensure a proper T cell response in tilapia.

### 2.8 mTORC1 Coordinates MAPK/ERK Signaling to Promote c‐Myc‐Mediated Glutaminolysis and T Cell Response in Tilapia

Next, we examined how TCR signaling initiates c‐Myc‐mediated glutaminolysis in tilapia T cells. Owing to the high consistency between inducible c‐Myc expression and mTORC1 or ERK1/2 activation upon TCR stimulation or bacterial infection (Figure S8A,B, Supporting Information), we explored the potential regulatory effects of these two signaling pathways on c‐Myc‐mediated glutaminolysis and T cell response in tilapia. Inhibition of mTORC1 activity by rapamycin impaired c‐Myc elevation during TCR stimulation or bacterial infection, and in turn diminished the upregulation of ASCT2, SNAT2, GLS1, and GLUD (Figure 6M,N; Figure S8C,D, Supporting Information). More precisely, the blockade of mTORC1 signaling reduced the inducible expression of c‐Myc and caused a marked deficiency of GLS1 in T cells (Figure 6O; Figure S8E, Supporting Information). Coincidentally, T cells lacking MAPK/ERK activity in trametinib‐treated tilapia exhibited reduced ability to upregulate c‐Myc and glutaminolysis‐related enzymes in response to TCR stimulation or bacterial infection (Figure 6P,Q; Figure S8F,G, Supporting Information), suggesting that MAPK/ERK signaling is also essential for c‐Myc‐mediated glutaminolysis in tilapia. Moreover, the loss of mTORC1 or MAPK/ERK activity inhibited T cell expansion during the primary immune response (Figure 6R,S; Figure S8H, Supporting Information) and reduced the production of IFN‐*γ* or perforin A in spleen lymphocytes (Figure 6T; Figure S8I, Supporting Information). To further prove that mTORC1 and MAPK/ERK signaling indeed exert their regulation on T cell through c‐Myc‐mediated glutaminolysis, *α*‐KG, a metabolite of glutamine was used to rescue the defective T cell response. Upon c‐Myc, mTORC1 or MAPK/ERK inhibition, addition of additional *α*‐KG partially rescued the dampened IL‐2 and TNF‐*α* expression during T cell activation (Figure S8J–L, Supporting Information) and the impaired T cell proliferation (Figure 6U). Therefore, these results suggest that downstream of TCR signaling, mTORC1 coordinates the MAPK/ERK pathway to promote c‐Myc‐mediated glutamine metabolism and T cell immune response in tilapia.

### 2.9 Glutamine Metabolism‐Driven T Cell Immunity is Conserved between Tilapia and Tetrapod

**Figure 7:**
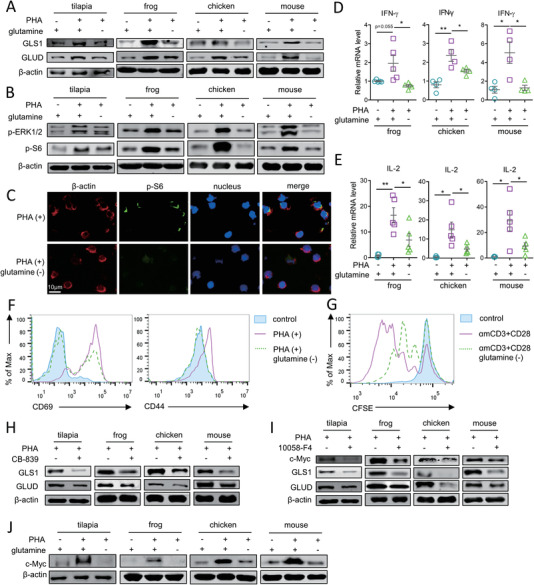
Different vertebrates share conserved regulatory mechanism of glutaminolysis on T cell immunity. Spleen leukocytes of tilapia, frog, chicken, or mouse were cultured in DMEM with or without glutamine, and stimulated with 2 µg mL^−1^ PHA. A) Protein levels of GLS1 and GLUD were examined at 24 h. Phosphorylation level of ERK1/2 or S6 were examined by B) western blot or C) immunofluorescence assay at 6 h. Relative mRNA levels of D) IFN‐*γ* and E) IL‐2 at 12 h after stimulation. F) Comparation of CD69 and CD44 expression in mouse spleen T cells at 12 h after stimulation. G) CFSE‐labeled mouse splenocytes were stimulated with 2 µg mL^−1^ CD3 and CD28 mAbs with or without glutamine, and T cell proliferation was examined at 72 h. Spleen leukocytes of tilapia, frog, chicken, or mouse were stimulated with 2 µg mL^−1^ PHA in the present or absent of H) 10 nM GLS1 inhibitor CB‐839 or I) 5 nM Myc inhibitor 10058‐F4 for 12 h and western blot shown protein levels of indicated molecules. J) Spleen leukocytes of tilapia, frog, chicken, or mouse were cultured in DMEM with or without glutamine, and stimulated with 2 µg mL^−1^ PHA. Protein level of c‐Myc was examined at 12 h after stimulation. These experiments were repeated for three independent times. *: *p* < 0.05, **: *p* < 0.01, determined by a two‐tailed Student's *t*‐test.

Our finding that the teleost Nile tilapia possesses a sophisticated strategy of using glutaminolysis to drive T cell response similar to that in mouse, supports the hypothesis that T cell immunity regulated by glutamine metabolism represents an evolutionarily conserved principle common to vertebrates. To verify this hypothesis, the correlation between T cell response and glutaminolysis was examined in various vertebrate lineages, including tilapia, frog (*X. laevis*), chicken, and mouse. In spleen leukocytes of tilapia, frog, chicken, or mouse, PHA‐induced T cell activation was associated with a marked elevation of GLS1 and GLUD, and glutamine deprivation impaired their inducible expression (**Figure** **7**A; Figure S9A, Supporting Information). Meanwhile, glutamine deprivation decreased the phosphorylation of ERK1/2 and S6 (Figure 7B,C; Figure S9B, Supporting Information), and downregulated IFN‐*γ* and IL‐2 expression (Figure 7D,E). In mouse T cells, glutamine deprivation impaired the upregulation of early activation markers CD69 and CD44 (Figure 7F), and compromised their proliferation (Figure 7G). GLS1 inhibition impaired GLUD expression and activity in PHA‐stimulated spleen leukocytes of tilapia, frog, chicken, and mouse (Figure 7H; Figure S9C,D, Supporting Information), and c‐Myc blockade reduced the glutaminolysis enzymes (Figure 7I; Figure S9E,F, Supporting Information), suggesting that these vertebrates employ the same, glutaminolysis‐dependent strategy to regulate T cell‐activation. Furthermore, glutamine deprivation in turn decreased c‐Myc expression during T cell activation; this effect was highly conserved across tilapia, frog, chicken, and mouse (Figure 7J), suggesting that the integration of transcription and glutamine metabolism to regulate T cell immunity may be a basic design principle in vertebrate evolution. Overall, these results indicate that the glutamine dependency of T cell activation and proliferation may be a common trait in bony vertebrates.

### 2.10 Rebuilding the Glutaminolysis Axis Using Tilapia Components Rescues the Immunodeficiency of Human T Cells

**Figure 8:**
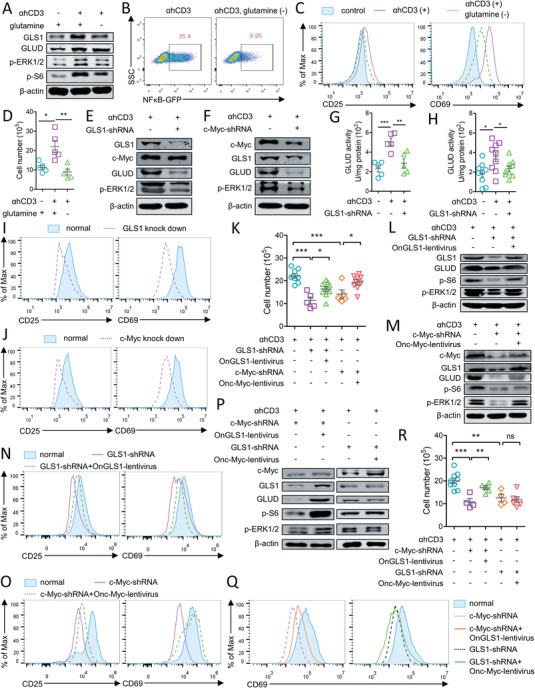
Rebuilding the glutaminolysis pathway with tilapia components restores immunodeficiency of human T cells. Jurkat cells cultured in the medium with or without glutamine were stimulated with 2 µg mL^−1^ CD3 mAb. A) Protein levels of indicated molecules at 12 h. B) NF*κ*B expression in NF*κ*B‐inducible GFP reporter Jurkat cells was shown by the GFP levels at 6 h. C) Comparation expression of CD25 and CD69 at 12 h. D) Cell numbers at 72 h after stimulation. Jurkat cells that transfected with control, GLS1 or c‐Myc‐shRNA were stimulated with 2 µg mL^−1^ CD3 mAb. E,F) Protein levels of indicated molecules at 12 h. G,H) GLUD activities at 12 h. I,J) Comparation expression of CD25 and CD69 at 12 h. GLS1 or c‐Myc‐interfered Jurkat cells were overexpressed with tilapia GLS1 or c‐Myc, and stimulated with 2 µg mL^−1^ CD3 mAb. K) Cell numbers at 72 h after stimulation. L,M) Protein levels of indicated molecules at 12 h. N,O) Comparation expression of CD25 and CD69 at 12 h. GLS1 or c‐Myc‐interfered Jurkat T cells were overexpressed with tilapia c‐Myc or GLS1, and stimulated with 2 µg mL^−1^ CD3 mAb. P) Protein levels of indicated molecules at 12 h. Q) Comparation expression of CD69 at 12 h. R) Cell numbers at 72 h after stimulation. These experiments were repeated for three independent times. *: *p* < 0.05, **: *p* < 0.01, ***: *p* < 0.001, determined by a two‐tailed Student's *t*‐test.

Conserved features of T cell immunity may guide the efforts to develop a synthetic immunological approach and provide novel avenues for intervention the defective immune functions in human. In this regard, an immortalized T cell line Jurkat was used to assess the conservation of the glutaminolysis dependency of T cell immunity between tilapia and human. Similar to the findings in tilapia, glutamine deprivation in Jurkat cells reduced the upregulation of GLS1 and GLUD upon TCR stimulation (**Figure** **8**A) and impaired T cell activation, as evidenced by the reduced ERK1/2 and S6 phosphorylation (Figure 8A), compromised NF‐*κ*B expression (Figure 8B), and diminished upregulation of T cell activation markers (Figure 8C). Moreover, lack of glutamine impaired the activation‐induced growth of Jurkat cells (Figure 8D). GLS1 interference by shRNA (Figure S10A, Supporting Information) impaired GLUD but not c‐Myc expression (Figure 8E); in contrast, c‐Myc knockdown caused defective GLS1 and GLUD expression (Figure S10A, Supporting Information; Figure 8F). Furthermore, RNA interference of GLS1 or c‐Myc markedly reduced ERK1/2 phosphorylation (Figure 8E,F), impaired GLUD activity (Figure 8G,H), and compromised antigen‐induced activation (Figure 8I,J) and growth (Figure 8K) of Jurkat cells. These findings indicate the potential existence of a conserved regulatory axis involving glutaminolysis and T cell response between tilapia and humans. To further examine whether the glutamine metabolism genes of tilapia may substitute for the corresponding components in humans, Jurkat cells lacking GLS1 or c‐Myc were infected with lentivirus expressing tilapia GLS1 or c‐Myc to restore the glutaminolysis regulatory axis (Figure S10B,C, Supporting Information). Introduction of tilapia GLS1 or c‐Myc into Jurkat cells evidently rescued the impaired expression of GLS1 and GLUD (Figure 8L,M), indicating the potential functionality of tilapia proteins in human cells. Notably, both tilapia GLS1 and c‐Myc were able to substitute for their human counterparts to rescue the defective activation signaling (Figure 8L,M), and in turn restore the impaired activation (Figure 8N,O) and proliferation (Figure 8K) of Jurkat cells. Furthermore, we revealed that overexpressing tilapia GLS1 in c‐Myc‐deficient Jurkat cells, but not tilapia c‐Myc in GLS1‐lacking Jurkat cells, was sufficient to rescue the compromised glutaminolysis (Figure 8P), activation (Figure 8P,Q), and expansion (Figure 8R) of these cells. Thus, our results suggest that a conserved mechanism is employed in tilapia and human to regulate glutaminolysis and ensure proper T cell response, and that the restoration of glutaminolysis regulatory axis with tilapia core components rescues the immunodeficiency of human T cells.

## 3 Discussion

Although T cell function in teleost have been widely studied in the past several decades, the current evidence is scattered across many fish species belonging to different genera, families, and even orders, and their inherent differences make it challenging to consolidate these findings to obtain a comprehensive landscape similar to that in mouse. Therefore, in the present study, we established several useful models in Nile tilapia to comprehensively study the T cell function and mechanism in this lower vertebrate. We found that tilapia T cells are involved in the primary and secondary immune response and exert cytotoxicity similar to their counterparts in rainbow trout and ginbuna crucian carp,^[^ ^30^, ^33^ ^]^ and that T cell inhibition or depletion in tilapia prevents adequate elimination of infection, causing high mortality. Given that amphibians also exhibit a CD8^+^ T cell response against viral infections,^[^ ^54^ ^]^ we speculate that using T cells to resist pathogen infections is an ancient strategy that developed early in vertebrate evolution.

T cell activation is delicately manipulated by sophisticated pathways in mouse.^[^ ^55^, ^56^, ^57^ ^]^ However, the mechanisms underpinning T cell activation in teleosts have remained largely unknown owing to the paucity of suitable models and procedures. In the present study, we established an in vitro T cell activation model in tilapia using CD3 and CD28 mAbs, which to our knowledge represents the first description of its kind in non‐mammals. With this approach, we illustrated the early activation events occurring downstream of TCR signaling, and revealed a regulatory network comprising the MAPK/ERK, Ca^2+^‐NFAT, NF‐*κ*B, and mTORC1 pathways that promotes T cell activation in tilapia. In addition, we speculate that the activation strategy dependent on the first and secondary signals used by mouse and human T cells^[^ ^58^, ^59^ ^]^ has existed since early in vertebrate evolution and thus has been programmed in the teleost Nile tilapia as well. These signaling pathways and strategies indicate adherence to a common design principle of T cell activation in tilapia and mammals, which to a large extent explains the functional similarity of T cells in these organisms. Notably, the artificial microenvironments provided by CD3 and CD28 mAb crosslinking enabled us to determine the molecular underpinnings of tilapia T cell priming, and demonstrate that the minimum prerequisites for T cell activation may be evolutionarily conserved and determined by molecular interactions rather than the structural environment.

The collaboration between T and B cells determines the initiation, maintenance, and outcome of the adaptive immune response. B cells provide the first and secondary signals essential for T cell activation,^[^ ^60^, ^61^ ^]^ and T cells facilitate the germinal center formation, Ig class switching and high‐affinity maturation in B cells.^[^ ^62^, ^63^ ^]^ Using Nile tilapia models in which T cells or IgM^+^ B cells can be selectively depleted during adulthood, we revealed that tilapia B cell expansion and antibody production need assistance from T cells, and B cells are in turn required for adequate T cell activation and function. These findings obtained using the selective depletion approach in tilapia, together with the observations made following KLH immunization in zebrafish,^[^ ^39^, ^64^ ^]^ collectively confirm the cooperation between T cells and B cells in different teleost species. In mammals, despite lymph nodes provide highly sophisticated tissue environments that promote the cellular interactions leading to T cell activation, T cells may be primed outside tissue‐draining lymph nodes like infected or inflamed tissue.^[^ ^65^ ^]^ The present findings indicate that such an activation approach may have originated in teleost, and it is reasonable to speculate that in animals lacking lymph nodes and germinal center, lymphoid organs such as spleen or head kidney provide the location necessary for T and B cell interaction. Given that no Tfh cells have been found in teleost, which subset of T cells plays a major role in assisting B cell response has become the next challenging question.

The immune system and metabolism are highly integrated, and multilevel interactions exist between metabolic reprogramming and T cell function and fate,^[^ ^66^, ^67^ ^]^ therefore nutrient availability and utilization are pivotal factors underpinning T cell immunity. In mouse, naïve T cells mainly use OXPHOS and FAO to generate ATP; however, upon activation, T cells switch their metabolic program to aerobic glycolysis and glutaminolysis, and enhance OXPHOS.^[^ ^66^, ^67^ ^]^ This reprogramming fulfills the demands of biomass accumulation and further promotes T cell growth, proliferation, and cytotoxicity. Notably, although the living environment and dietary structure of tilapia and mouse are markedly different, their T cells exhibit similar metabolic reprogramming. In fact, T cell is not the unique cell lineage that switches the metabolism during immune response of teleost, because M1 and M2 carp macrophages show distinct metabolic signatures and a metabolic reprogramming may occur in M1 macrophages.^[^ ^68^ ^]^ Considering tilapia also utilizes a set of evolutionarily conserved transcription factors to regulate the T cell response, we speculate that the conservative transcriptional networks and metabolic programs underlie the functional similarities of T cells between tilapia and mouse.

Metabolic reprogramming characterized by the increased consumption of glutamine has always been emphasized in T cell immunity.^[^ ^69^ ^]^ Glutaminolysis provides essential raw materials and intermediates for anabolic pathways to ensure proper T cell proliferation and effector function.^[^ ^10^, ^70^ ^]^ Additionally, glutamine metabolites reinforce T cell immunity through epigenetic remodeling or maintenance of the redox balance.^[^ ^12^, ^13^ ^]^ In the present study, T cell activation was accompanied by increased glutamine uptake and utilization, whereas glutamine deprivation or GLS1 inhibition markedly impaired T cell activation, proliferation, and function in tilapia. In addition, we confirmed the presence of a sophisticated mechanism downstream of TCR signaling, by which mTORC1 coordinates MAPK/ERK pathway to promote c‐Myc‐mediated glutaminolysis and T cell response, indicating that c‐Myc integrates immune signals and metabolic programs through transcriptional regulatory networks. Remarkably, at the same time of c‐Myc‐inhibition blocks glutamine metabolism in tilapia T cells, glutamine deprivation also weakens c‐Myc expression through feedback regulation, suggesting the existence of a regulatory mechanism by which activation‐induced transcriptional and metabolic networks communicate in a bidirectional manner to determine the T cell response. These findings support a notion that the integration of T cell response and metabolism is an evolutionarily ancient strategy that had already emerged in some teleost species. However, whether glutamine metabolism regulates tilapia T cell immunity through bioenergy supply, building block biosynthesis, redox maintenance, or epigenetic modifications remain unclear. Whether other transcription factors such as c‐Jun or HIF‐1*α* ^[^ ^71^, ^72^ ^]^ modulate glutaminolysis in tilapia T cells is another question that needs further investigation. Furthermore, the effects of specific inhibitors on other cell lineages cannot be excluded. In future studies, knockout models based on LCK or CD4 Cre‐driven CRISPR/Cas9 are expected to elucidate the intrinsic role of glutamine metabolism in T cell response.

The communication between metabolism and immunity is reciprocal and rooted in the objective of conferring more survival advantages. Remarkably, T cell immunity in tilapia, frog, chicken, and mouse, which belong to different vertebrate lineages with dissimilar immune organs, living environments, and diets, exhibits the same dependence on glutamine metabolism and shares an identical regulatory pathway. Of note, to simultaneously activate T cells of frog, chicken, and mouse, the PHA was employed in present study. Although this cannot completely exclude the potential effect of macrophages with similar metabolic reprogramming properties, such interference should not be predominant. Because as a wide accepted T cell mitogen,^[^ ^73^ ^]^ PHA activates T cells more effectively than other cell lineages.^[^ ^73^, ^74^ ^]^ Our findings further confirm the conclusion that the metabolic dependency of T cell response did not occur spontaneously in mammals, but had already emerged in some teleost species and may be a strategy conserved throughout vertebrate evolution. Since pathogens, environment, transcription, and nutrients are all potential factors affecting the evolution of the immune system, the similarities in immune signals, metabolism programs, and transcriptional networks between tilapia and other vertebrates identified in the present study largely explain the functional similarities of T cells in these organisms. In addition, we found that glutamine deprivation or GLS1 knockdown markedly impaired the activation and growth of human Jurkat T cells. Benefiting from the above‐mentioned functional similarities, overexpression of the corresponding tilapia proteins in GLS1‐deficient or c‐Myc‐deficient Jurkat cells significantly rescued the impairment of cell activation and growth. Considering that both GLS1 and c‐Myc are potential targets for the therapy of cancer or immune disorders,^[^ ^75^, ^76^ ^]^ our findings would be helpful to understand the cancer progression and immune reconstruction.

In summary, we provided a comprehensive landscape of T cell response in the teleost Nile tilapia (Figure S11, Supporting Information). Upon bacterial infection, antigen‐presenting cells (i.e., IgM^+^ B cells and macrophages) provide the first and secondary signals for T cells, initiating the Ca^2+^‐NFAT, MAPK/ERK, NF‐*κ*B, and mTORC1 pathways, leading to IL‐2 transcription and T cell activation. In turn, the MAPK/ERK and mTORC1 pathways trigger the c‐Myc‐mediated glutamine metabolism to ensure proper T cell activation, proliferation, and cytotoxicity. Moreover, effector T cells interact with IgM^+^ B cells to facilitate the humoral immune response. Upon antigen clearance, a majority of effector T cells die by apoptosis, and the remaining small proportion of T cells form cellular memory response. From an evolutionary viewpoint, the mechanism of the maintenance and regulation of T cell response by glutamine metabolism is highly conserved across diverse vertebrate lineages such as tilapia, frog, chicken, and mouse, implying its potential role in supporting the functional similarities of T cells in vertebrates. Notably, benefiting from this functional conservation, reconstruction of the glutaminolysis pathway using tilapia components can rescue the immunodeficiency of human T cells. Our findings thus illustrate the underlying mechanism of T cell immunity in teleost, and provide novel perspectives to understand the evolution of adaptive immune system.

## 4 Experimental Section

### Animals

Nile tilapia *Oreochromis niloticus* were purchased from an aquaculture farm in Guangzhou, Guangdong Province, China. All fish were maintained in recirculating water at 28 °C, and with daily feeding for at least 2 weeks before experiments began. Healthy fish ≈8–10 cm in length were used for experiments. *Gallus gallus* and *Xenopus laevis* were purchased from a Laboratory Animal Breeding Center in Shanghai, China, and were kept in animal facilities at the East China Normal University. BALB/c mice and rats were bred and housed in the same facilities. All animal care and experimental procedures were performed in accordance with the Guide for the Care and Use of Laboratory Animals of the Ministry of Science and Technology of China and were approved by the East China Normal University Experimental Animal Ethics Committee (no. AR2021‐245). All efforts were made to minimize the pain of animals.

### Cell Lines

HEK293T, BOSC23, NIH/3T3, and Jurkat cells were purchased from the American Type Culture Collection (ATCC, Manassas, VA, USA). The mouse myeloma SP2/0 was kindly donated by the Ocean University of Shanghai, China. The Jurkat NF‐*κ*B‐green fluorescent protein (GFP) reporter cells were generated in the previous study.^[^ ^77^ ^]^

### Bioinformatics Analysis

The cDNA and amino acid sequences were obtained from the National Center for Biotechnology Information (NCBI) GenBank (https://www.ncbi.nlm.nih.gov) and analyzed by BLAST. The functional domains of proteins were predicted by SMART (http://smart.embl‐heidelberg.de) and illustrated using DOG 2.0. Tertiary structures were predicted by the SWISS‐MODEL and displayed in PyMOL. The multiple sequence alignment was performed using the Clustal X 1.83, and phylogenetic tree was constructed using the neighbor‐joining method with the MEGA program. The accession numbers of all sequences used are listed in Table S2, Supporting Information.

### Bacterial Infection

Nile tilapia individuals were intraperitoneally (i.p.) injected with 100 µL of 1 × 10^7^, 4 × 10^7^, 8 × 10^7^, or 4 × 10^8^ CFU mL^−1^ of *E. piscicida* isolated from turbot^[^ ^78^ ^]^ to explore the appropriate dose. For subsequent bacterial infection, tilapia individuals were injected with 100 µL of 4 × 10^7^ CFU mL^−1^ of *E. piscicida*, and the control animals were injected with the same volume of phosphate‐buffered saline (PBS). During infection, fish were i.p. injected with different kinds of inhibitors or not injected, and leukocytes were isolated on indicated days for assays. For survival analysis, animal mortality in each group was recorded each day and finally calculated. For bacterial titer analysis, liver or spleen were harvested and grinded on the indicated days, and bacterial load was calculated by counting the colonies that appeared on the Luria‐Bertani (LB) agar plates.

### Inhibitor Treatment

For the in vivo inhibition assay, bacterially infected or uninfected Nile tilapia were i.p. injected with a specific inhibitor on indicated days described in the Figure legends, and the control animals were injected with the same volume of PBS. The dose per injection of inhibitors were as follows: Cyclosporine A (Catalog #HY‐B0579, MedChemExpress), 10 mg kg^−1^; GLS1 inhibitor CB‐839 (Catalog #HY‐12248, MedChemExpress), 20 mg kg^−1^; c‐Myc inhibitor 10058‐F4 (Catalog #HY‐12702, MedChemExpress), 10 mg kg^−1^; mTORC1 inhibitor rapamycin (Catalog #HY‐10219, MedChemExpress), 1 mg kg^−1^; and Mek inhibitor Trametinib (Catalog #HY‐10999, MedChemExpress), 0.1 mg kg^−1^. Animals were sacrificed on the indicated days for assays. For the in vitro inhibition assay, spleen leukocytes of Nile tilapia, frog, chicken, and mouse were cultured in Dulbecco's modified eagle medium (DMEM) containing 10% fetal bovine serum (FBS) and 1% penicillin/streptomycin in the presence of the indicated inhibitor. The inhibitors were used at the following concentrations: Cyclosporine A, 1 nM; CB‐839, 10 nM; 10058‐F4, 5 nM; rapamycin, 10 nM; Trametinib, 10 nM; NF‐*κ*B inhibitor QNZ (Catalog #HY‐13812, MedChemExpress) 10 nM. To rescue the glutaminolysis defect‐impaired T cell response, 1 mm *α*‐KG (Catalog #A610290‐0100, BBI Life Sciences) was added into the culture medium of tilapia spleen leukocytes in the presence of 10058‐F4, rapamycin, or trametinib.

### Leukocyte Isolation

Leukocytes were isolated from various tissues of Nile tilapia. The spleen and head kidney were harvested, washed, and grinded in pre‐cooled Leibovitz's L‐15 medium (Catalog #41300‐039, Gibco), and cell suspensions were filtered using a nylon mesh. Peripheral blood was withdrawn from the caudal vein, immediately mixed with an anticoagulant, and resuspended with L‐15 medium after centrifugation. These cell suspensions were layered onto 52% and 34% Percoll (Catalog #10 246 712, GE Healthcare) density gradients, and centrifugated at 500 × *g* at room temperature for 35 min. Leukocytes present at the interface of the discontinuous gradients were collected, washed, and resuspended with DMEM containing 10% FBS. Spleen tissue was harvested from *G. gallus*, *X. laevis*, or mouse. After lysis of red blood cells with the ACK buffer, splenocytes were resuspended in DMEM (10% FBS, 1% penicillin and streptomycin) for further use.

### Cell Stimulation

For T cell activation, 1 × 10^6^ spleen leukocytes of Nile tilapia cultured in DMEM (10% FBS, 1% penicillin and streptomycin) were stimulated with anti‐tilapia CD3 (2 µg mL^−1^) for indicated time points. For T cell proliferation assay, 1 × 10^6^ spleen leukocytes of Nile tilapia were stimulated using plate‐bound anti‐tilapia CD3 (2 µg mL^−1^) plus soluble anti‐tilapia CD28 (2 µg mL^−1^) for 48 or 72 h. 1 × 10^6^ mouse splenocytes were stimulated with 2 µg mL^−1^ of anti‐mouse CD3 (Catalog #100 301, BioLegend) plus anti‐mouse CD28 (Catalog #102 101, BioLegend) for 72 h. 1 × 10^6^ Jurkat cells cultured in RPMI‐medium were stimulated with 2 µg mL^−1^ of anti‐human CD3 (Catalog #300 302, BioLegend) for indicated time points. Splenocytes of tilapia, *G. gallus*, *X. laevis* or mouse were activated by 2 µg mL^−1^ of PHA (Catalog #L4144, Sigma). For stimulation under glutamine starvation, spleen leukocytes isolated from tilapia, *G. gallus*, *X. laevis*, or mouse were precultured in the medium without glutamine for 6 h, and were then subjected to indicated stimulation. Jurkat cells were precultured in the medium without glutamine for 12 h, and were then subjected to indicated stimulation for 6 h to detect NF‐*κ*B expression or for 12 h to detect the expression of indicated molecules. For Jurkat proliferation assay, 1 × 10^5^ cells were stimulated by 2 µg mL^−1^ anti‐human CD3 in the presence or absence of glutamine for 72 h.

### IgM Purification

Soluble IgM of Nile tilapia was purified from serum using protein A agarose (Invitrogen) according to manufacturer's instructions. In brief, the serum was incubated with protein A agarose at 4 °C overnight. After being washed with pre‐cooled PBS, IgM was eluted with 0.1 m glycine‐HCl (pH 2.8) and neutralized with 1 m Tris‐HCl (pH 8.5). After dialysis in PBS, purified IgM was subjected to SDS‐PAGE and stained with Coomassie blue. Protein bands for suspect heavy and light chains of IgM were cut for matrix‐assisted laser desorption/ionization time‐of‐flight mass spectrometry by Sangon Biotech (China). The resulting spectra were used as query to search the NCBI non‐redundant database.

### Retrovirus Packaging and Cell Infection

The full‐length coding region of CD3*ε* or CD28 was amplified from Nile tilapia cDNA and cloned into the vector Migr1. 2 × 10^6^ BOSC23 packaging cells were seeded in 6 cm dishes overnight. Then, BOSC23 cultured in DMEM containing 25 nM chloroquine (Catalog #C6628, Sigma) were transfected with 5 µg pCL‐Eco plus 5 µg Migr1‐CD3*ε* or Migr1‐CD28 plasmids dissolved in HEBS buffer (50 mm HEPES, 10 mm KCl, 12 mm Dextrose, 280 mm NaCl, 1.5 mm Na_2_HPO4, pH 7.05) containing 0.1 mm CaCl_2_ for 8 h. After replacing the medium with fresh one, the supernatant was collected at 48 h and centrifuged at 1200 rpm for 7 min to obtain the retrovirus. 120 µL of retrovirus was used to infect 2 × 10^5^ NIH/3T3 cells pre‐seeding in 6 cm dishes overnight in the presence of 5 µg mL^−1^ polybrene (Catalog #H9268, Sigma). After adding 500 µL fresh medium to dilute the polybrene at 5 h post infection, the culture supernatant was replaced with polybrene‐free medium on the following day. Infected NIH/3T3 cells were harvested at 48 h for GFP detection and mouse immunization.

### Development of Monoclonal and Polyclonal Antibodies against Nile Tilapia CD3*ε*, CD28, and IgM

For IgM mAb preparation, BALB/c mice were first immunized by intraperitoneal injection with 100 µg purified IgM emulsified in complete Freund's adjuvant. 2 weeks later, another intraperitoneal injection with the same quantity of antigen in incomplete Freund's adjuvant was administered, followed by two booster immunizations with 100 µg IgM in PBS via the tail vein at 1‐week intervals. To develop CD3*ε* or CD28 mAb, mice were i.p. immunized with 2 × 10^7^ CD3*ε*‐NIH/3T3 or CD28‐NIH/3T3 cells four times with the same intervals as above. 3 days after the last immunization, the spleen of immunized mice was harvested for cell fusion with SP2/0 myeloma cells using 50% polyethylene glycol 4000. The fused cells were cultured in RPMI medium containing 10% FBS and 1% HAT (Catalog #21 060 017, Gibco). After 7–10 days, supernatants of the hybridomas were screened by ELISA, flow cytometry, immunofluorescence, or western blot. After confirmation by cell sorting and gene expression assays, positive hybridomas were cloned using the limiting dilution method. The hybridomas confirmed to be positive for Nile tilapia IgM, CD28, or CD3*ε* were i.p. injected into BALB/c mice to induce ascites, and mAbs were purified from ascites using protein G agarose (Invitrogen) and labelled with FITC (Catalog #F3651, Sigma) or biotin (Catalog #H1759, Sigma) for further experiments. To generate polyclonal antibodies against Nile tilapia IgM, rats were immunized with 100 µg purified IgM as above. The animals were sacrificed 3 days after the last immunization to obtain the anti‐serum, and polyclonal antibodies were purified from the serum using protein G agarose.

### ELISA Assay

To identify hybridomas positive for Nile tilapia IgM, a 96‐well plate was coated with 2 µg mL^−1^ purified IgM in 50 mm carbonate–bicarbonate buffer (pH 9.6) at 4 °C overnight and then was blocked with 2% bovine serum albumin (BSA) at 37 °C for 1 h. After washing the plate with PBST three times, 100 µL of hybridoma supernatant was added to the well and incubated at room temperature for 1 h. The plate was then washed as above; 100 µL alkaline phosphatase‐conjugated goat anti‐mouse IgG H&L (Catalog #710‐1502, Southern Biotech, 1:4000) was added, and the plate was incubated for another 1 h. The plate was again washed thrice with PBST, and 100 µL of 0.1% p‐nitrophenyl phosphate (Catalog #4876, Sigma) in 50 mm carbonate–bicarbonate buffer (pH 9.8) containing 0.5 mm MgCl_2_ was added; the plate was incubated in the dark. The reaction was finally stopped by adding 50 µL of 2 m NaOH, and the absorbance was measured at 405 nm. The supernatant of SP2/0 myeloma was used as a negative control. When examining the IgM levels in Nile tilapia serum, 1:50 diluted serum was added to the 96‐well plate precoated with 2 µg mL^−1^ rat‐anti‐IgM polyclonal antibody, and the IgM mAb generated in this study was used as the primary antibody.

### Western Blot

1 × 10^7^ freshly isolated or in vitro stimulated leukocytes were lysed with NP40 lysis buffer (1% NP40, 150 mm NaCl, 50 mm Tris; pH 7.4) on ice for 30 min. The supernatants with protein concentration of about 1.5 mg mL^−1^ were then subjected to SDS‐PAGE and transferred to a nitrocellulose membrane. The membranes were blocked with 4% milk powder at room temperature for 1 h and incubated with antibodies for p‐LCK (Tyr505, Catalog #2751, Cell Signaling Technology, 1:1000), p‐ZAP70 (Tyr493, Catalog #2704, Cell Signaling Technology, 1:1000), p‐PLC*γ*1 (Tyr783, Catalog #2821, Cell Signaling Technology, 1:1000), p‐ERK1/2 (Thr202/Tyr204, Catalog #4370, Cell Signaling Technology, 1:1000), p‐JNK (Thr183/Thr183/Thr221, Catalog #AF1762, Beyotime, 1:1000), p‐S6 (Ser240/244, Catalog #5364, Cell Signaling Technology, 1:1000), p‐NF*κ*B p65 (Ser536, Catalog #AF5881, Beyotime, 1:1000), p‐mTOR (Ser2448, Catalog #5536, Cell Signaling Technology, 1:1000), PLC*γ*1 (Catalog #5690, Cell Signaling Technology, 1:1000), ZAP70 (Catalog #3165, Cell Signaling Technology, 1:1000), ERK1/2 (Catalog #4695, Cell Signaling Technology, 1:1000), JNK (Catalog #AF1048, Beyotime, 1:1000), S6 (Catalog #2217, Cell Signaling Technology, 1:1000), NF*κ*B p65 (Catalog #AF0246, Beyotime, 1:1000), CaM (Catalog #4830, Cell Signaling Technology, 1:1000), GLS1 (Catalog #49 363, Cell Signaling Technology, 1:1000), GLUD (Catalog #12 793, Cell Signaling Technology, 1:1000), c‐Myc (Catalog #13 871, Cell Signaling Technology, 1:1000), and *β*‐actin (Catalog #4970, Cell Signaling Technology, 1:1000) at 4 °C overnight. The blots were washed three times with PBST and further incubated with goat anti‐rabbit IgG H&L Alexa Fluor 800 (Catalog #5151, Cell Signaling Technology, 1:30 000) or goat anti‐mouse IgG H&L Alexa Fluor 680 (Catalog #ab175775, Abcam, 1:10 000) at room temperature for 1 h. The blots were washed again three times and scanned using Odyssey CLx Image Studio. The interaction of the mAb generated herein with purified serum IgM or spleen leukocyte lysate was analyzed as above, and incubated with hybridoma supernatant as primary antibody. Binding was detected with goat anti‐mouse Ig–alkaline phosphatase conjugate (Catalog #710‐1502, Southern Biotech, 1:4000) and developed with freshly prepared substrate solution (100 mm NaCl, 100 mm Tris, and 5 mm MgCl_2_; pH 9.5) containing nitroblue tetrazolium (Catalog #11 585 029 001, Sigma) and 5‐bromo‐4‐chloro‐3‐indolylphosphate (Catalog #B6149, Sigma).

### Immunofluorescence

1 × 10^6^ transfected cells and spleen leukocytes that treated with CD3 mAb in the presence or absence of glutamine, or leukocytes incubated with FITC‐labelled *E. piscicida*, were spun onto the slides by Cytospin (Thermo). All samples were fixed in prechilled acetone for 15 min and blocked with 1% BSA at room temperature for 1 h. To detect CD3‐GFP expression on the membrane NIH3T3 cells, the cells was stained with 5 µM Dil (Catalog #C1036, Beyotime) at 37 °C for 5 min and washed twice with PBS. To detect the expression of CD3 or IgM on leukocytes, the samples were stained using CD3*ε* or IgM hybridoma supernatant as primary antibody and FITC‐conjugated goat‐anti‐mouse IgG H&L (Catalog #ab6785, Abcom, 1:800) as secondary antibody. To evaluate ERK1/2 or S6 phosphorylation in leukocytes, 1 × 10^6^ cells stimulated with 2 µg mL^−1^ CD3*ε* mAb or 2 µg mL^−1^ PHA were cultured in DMEM with or without glutamine for 6 h, and then were stained using anti‐*β*‐actin (Catalog #3700, Cell Signaling Technology, 1:2400), p‐ERK1/2 (Thr202/Tyr204, Catalog #4370, Cell Signaling Technology, 1:200), or p‐S6 (Ser240/244, Catalog #5364, Cell Signaling Technology, 1:800) as primary antibody, and Alexa Fluor 594‐conjugated goat‐anti‐rabbit IgG H&L (Catalog #ab150080, Abcam, 1:800), Alexa Fluor 594‐conjugated Goat Anti‐Mouse IgG H&L (Catalog #ab150116, Abcam, 1:800), or FITC‐conjugated goat‐anti‐mouse IgG H&L (Catalog #ab6785, Abcom, 1:800) as secondary antibody. To detect the IgM coated on bacteria or phagocytosis of IgM^+^ B cells, bacteria were labelled with FITC, whereas IgM on bacteria or leukocytes was stained with IgM mAb followed by Alexa Fluor 594‐conjugated Goat Anti‐Mouse IgG H&L (Catalog #ab150116, Abcam, 1:800). All incubations were performed at room temperature for 1 h, and the samples were washed with PBS three times after each incubation. The cells were stained with Hoechst 33 342 and observed by fluorescence microscopy.

### Flow Cytometry and Cell Sorting

To identify the hybridomas positive for CD3, CD28, or IgM, 1 × 10^6^ spleen leukocytes were incubated with hybridoma supernatants on ice for 30 min. The cells were washed with FACS buffer (PBS containing 2% FBS) and further stained with 1:2000 diluted Alexa Fluor 647‐conjugated goat anti‐mouse IgG H&L (Catalog #ab150115, Abcam, 1:2000) on ice for another 30 min. To determine the expression of CD3 and IgM at the same time, cells were stained with FITC‐labelled CD3*ε* mAb and biotin‐labelled IgM mAb and followed by APC‐ or PE‐streptavidin (BioLegend) staining. To stain T cell activation markers, mouse splenocytes or Jurkat cells were incubated with antibodies for mouse CD44 (Catalog #103 011, Biolegend, 1:400), CD69 (Catalog #104 513, Biolegend, 1:400), or human CD25 (Catalog #302 605, Biolegend, 1:400), CD69 (Catalog #310 905, Biolegend, 1:400) on ice for 20 min. For intracellular p‐S6 and GLS1 staining, cells were first stained with CD3*ε* or IgM mAb as above and then fixed with the fixation/permeabilization solution (Catalog #554 722, BD Biosciences) on ice for 30 min. These cells were then washed with the Perm/Wash solution (Catalog #554 723, BD Biosciences) twice and further stained with 1:200 diluted PE‐conjugated anti‐p‐S6 Ser240/244 (Catalog #369 521, Biolegend, 1:200), or anti‐GLS1 (Catalog #49 363, Cell Signaling Technology, 1:400) followed by Alexa Fluor 647‐conjugated goat anti‐rabbit IgG H&L (Catalog # ab150079, Abcam, 1:2000), on ice for 30 min. For intracellular p‐ERK1/2, p‐NF‐*κ*B p65 or c‐Myc staining, leukocytes were fixed with Foxp3 Staining Buffer Set (Catalog #1 993 879, eBioscience) on ice for 2 h and further stained with APC‐conjugated anti‐p‐ERK1/2 Thr202/Thy204 (Catalog #935 703, Biolegend, 1:200), APC‐conjugated anti‐p‐NF*κ*B p65 Ser536 (Catalog #4887, Cell Signaling Technology, 1:200), or Alexa Fluor 647‐conjugated anti‐c‐Myc (Catalog #13 871, Cell Signaling Technology, 1:200) on ice for 30 min. All the stained cells were resuspended with FACS buffer and analyzed by BD FACSCanto II flow cytometer. Data were analyzed using FlowJo software. To sort CD3*ε* ^+^ T cells or IgM^+^ B cells, spleen leukocytes were stained with CD3*ε* or IgM mAb in DMEM containing 5% FBS as above, and the cells were resuspended in the same medium and sorted with a BD FACSAria II flow cytometer.

### RNA‐Seq Analysis

4 × 10^6^ naïve or effector T cells from healthy or *E. piscicida*‐infected tilapia at 5 DPI were sorted for RNA‐seq analyses. Total RNA was extracted using Trizol reagent kit (Catalog #15 596 018, Invitrogen) according to the manufacturer's protocol, and subjected to commercial RNA‐seq analyses (Gene Denovo Biotechnology) on an Illumina NovaSeq 6000 instrument with 150‐bp paired‐end reads. Reads were mapped to the Nile tilapia genome (Ensembl release100) with HISAT2 (v2.2.4). RNA‐seq was analyzed using standard methods on the platform of Omicsmart (http://www.omicsmart.com), including alignment to the genome using HISAT2, gene expression values by DESeq2 software between two different groups (and by edgeR between two samples), GO or Pathway enrichment analysis, Gene Set Enrichment Analysis using software GSEA, and MsigDB to display specific GO terms/KEGG pathways.

### Apoptosis Assay

1 × 10^6^ spleen leukocytes were first stained with FITC‐labelled CD3*ε* mAb as above. The cells were then washed with FACS buffer and stained with APC‐conjugated Annexin V antibody (Catalog #640 920, BioLegend, 1:400) in Annexin V binding buffer (0.01 m HEPES/NaOH, pH 7.4; 0.14 m NaCl, 2.5 mm CaCl_2_) at room temperature for 15 min. 7‐aminoactinomycin D (7‐AAD; Catalog #A1310, Invitrogen, 1:400) was added immediately before the samples were analyzed by flow cytometry.

### BrdU Incorporation

Control or *E. piscicida*‐infected Nile tilapia was i.p. injected with 0.75 mg BrdU (Catalog #19‐160, Sigma) in 200 µL PBS 1 day before the animals were sacrificed, and spleen leukocytes were isolated 24 h later for the assay. 2 × 10^6^ spleen leukocytes were first stained for surface CD3*ε* and IgM as above. The leukocytes were fixed with BD Cytofix/Cytoperm Buffer on ice for 30 min and washed twice with BD Perm/Wash Buffer. Subsequently, the cells were treated with BD Cytoperm Plus Buffer for 10 min and BD Cytofix/Cytoperm Buffer for 5 min on ice, and then digested with 300 µg mL^−1^ DNase at 37 °C for 1 h and washed with BD Perm/Wash Buffer. Samples were subsequently stained with 1:100 diluted FITC‐anti‐BrdU antibody (Catalog #51‐33284X, BD) at room temperature for 20 min and analyzed with flow cytometry.

### In Vitro Proliferation Assay

1 × 10^6^ spleen leukocytes were labelled with 10 µM CFSE (Catalog #C34554, Invitrogen) at room temperature according to the manufacturer's protocol. The labelled cells were cultured in DMEM containing or lacking glutamine at 28 °C for 48 or 72 h, and 2 µg mL^−1^ of CD3*ε* mAb and CD28 mAb were used to induce T cell proliferation. Cells were then stained with CD3*ε* mAb as above, and 7‐AAD was added to identify live/dead cells shortly before flow cytometry assay.

### Bacterial Coating Assays

To determine the ability of serum IgM to coat microbes, serum was obtained from *E. piscicida*‐infected Nile tilapia on indicated days. *E. piscicida* was treated at 65 °C for 1 h and subsequently inactivated in 4% paraformaldehyde at 4 °C for 24 h. The inactivated *E. piscicida* was incubated with 0.02 g mL^−1^ FITC (Catalog #F3651, Sigma) in 0.1 m NaHCO_3_ at 37 °C for 30 min. After washing with PBS, 5 µL control or specific serum was added into the bacteria suspension and incubated at room temperature for 2 h. The samples were then subjected to an immunofluorescence assay to detect the coated IgM on microbes using IgM mAbs. To quantify the binding of IgM to microbes, *E. piscicida* was incubated with serum from control or *E. piscicida‐*infected fish at room temperature for 2 h, and then western blotting was performed to examine the interaction using IgM mAbs.

### Phagocytosis Assay

To assess the opsonization function of IgM, FITC‐labelled *E. piscicida* was incubated with serum from control or *E. piscicida‐*infected fish at room temperature for 2 h. The microbes were washed with PBS, added to a suspension of 1 × 10^6^ head kidney leukocytes, and cultured at 28 °C for another 6 h. After that, 0.2% trypan blue was added to quench the fluorescence from attached bacteria. Phagocytosis of splenic IgM^+^ B cells or CD3^+^ T cells was then examined by flow cytometry or immunofluorescence after staining with IgM or CD3*ε* mAb. Myeloid cells were gated by flow cytometry according to the previous report.^[^ ^43^ ^]^ To inhibit the phagocytosis, 0.08 mg mL^−1^ cytochalasin B (Catalog #C6762, Sigma) was added to the culture medium.

### Cytotoxicity Assay

To examine the cytotoxicity of effector T cells, we i.p. injected the Nile tilapia individuals with 1 × 10^7^ 293T cells on day 1 and day 3. The animals were treated with indicated inhibitors on day 1, 3, and 5, and spleen T cells were sorted on day 6 for the assay. 2 × 10^6^ T cells were incubated with 2 × 10^5^ 293T cells or NIH3T3 cells at 28 °C for 8 h. Cytotoxicity was assessed as LDH release, using the Cytotoxicity Detection Kit Plus (Catalog #4 744 926 001, Sigma) according to the manufacturer's instructions.

### Ca^2+^ Influx Assay

2 × 10^6^ spleen leukocytes in loading buffer (1% FBS and 10 mm HEPES in HBSS without phenol red) were incubated with 2.5 mmol L^−1^ Indo‐1 (Catalog #I1203, Life Technologies) in the presence of Pluronic at 30 °C for 30 min. The cells were washed with loading buffer, and collected on a BD cytometer to obtain the baseline fluorescence of the 450/510 nm ratio on the lymphocyte population. Then, 2 µg mL^−1^ tilapia CD3 mAb was added to trigger Ca^2+^ influx. Subsequently, 2 µg mL^−1^ thapsigargin was added to induce maximal Ca^2+^ influx.

### Detection of DAG Concentration

1 × 10^6^ spleen leukocytes of tilapia that stimulated with 2 µg mL^−1^ of CD3 mAb for 45 min or not were disrupted by ultrasonication. The supernatants were then collected, and the DAG concentration was examined with commercial assay kits (Shanghai Fantbio) according to manufacturer's instructions.

### T or IgM^+^ B Cell Depletion

The T or IgM^+^ B cell depletion was determined according to a previous report with modification.^[^ ^79^ ^]^ Nile tilapia individuals were i.p. injected with 20 µg of mouse anti‐tilapia CD3*ε* or IgM mAb, or mouse IgG1 (Catalog #401 402, BioLegend) as isotype control. Then, 24 h later, the tilapia was injected with 50 µL tilapia anti‐mouse IgG1 serum or negative serum for the control group. Nine days after mAb injection, spleen leukocytes were isolated to determine the frequency of CD3*ε* ^+^ T cells and IgM^+^ B cells. The T cell‐ or IgM^+^ B cell‐depleted tilapia was then i.p. injected with *E. piscicida*, and animals were sacrificed on indicated days for the assay.

### Activation of T Cells by IgM^+^ B Cells

*E. piscicida* was treated at 65 °C for 1 h and subsequently inactivated in 4% paraformaldehyde at 4 °C for 24 h. T cells and IgM^+^ B cells were sorted from one same tilapia individual for assay. 1 × 10^6^ IgM^+^ B cells were incubated with 5 × 10^6^ CFU mL^−1^ of inactivated *E. piscicida* for 6 h. For T cell activation assay, 1 × 10^6^ sorted T cells were co‐cultured with the *E. piscicida*‐loaded or unloaded IgM^+^ B cells in 48‐well plate. Phosphorylation of ERK1/2, S6, and NF‐*κ*B p65 in T cells was examined at 8 h by flow cytometry. For T cell proliferation, 1 × 10^6^ CFSE‐labelled T cells were co‐cultured with the *E. piscicida*‐loaded or unloaded IgM^+^ B cells as above, and the CFSE dilution of T cells was determined at 48 h by flow cytometry.

### Evaluation of Glutamate Concentration

1 × 10^6^ spleen leukocytes of Nile tilapia that stimulated with 2 µg mL^−1^ of CD3 mAb with or without the indicated inhibitors for 24 h were disrupted by ultrasonication. The supernatants were then collected, and glutamate concentration was measured with commercial assay kits (Catalog #A074‐1‐1, Nanjing Jiancheng Bioengineering) according to manufacturer's instructions.

### Examination of Enzyme Activity

1 × 10^6^ spleen leukocytes isolated from Nile tilapia, *G. gallus*, *X. laevis* or mouse, or Jurkat cells were stimulated with 2 µg mL^−1^ of CD3 mAb or PHA in the presence or absence of glutamine or inhibitors for 12 or 24 h, and were disrupted by ultrasonication. Activity of GLUD was measured with commercial assay kits (Catalog #A125‐1‐1, Nanjing Jiancheng Bioengineering) according to manufacturer's instructions. The enzyme activity per mg of cell protein was calculated.

### qPCR Assay

Total RNA was extracted from freshly harvested or in vitro stimulated spleen leukocytes with TRIzol reagent (Cat #15 596 018, Invitrogen), and reverse‐transcribed with Plus All‐in‐one First‐Strand cDNA Sythesis SuperMix (Catalog #E096‐1B, Novoprotein). The 1:50 diluted cDNA was used as the template for the SYBR Green fluorescent real‐time qPCR assay. Expression levels of target genes were normalized with *β*‐actin and calculated using the 2^−△△CT^ method. All gene‐specific primers used in this study are listed in Table S3, Supporting Information.

### Dual‐Luciferase Assay

Tilapia IL‐2 promoter (−2000 to 0 bp) was cloned into pGL3 vector, and the coding regions of NF‐*κ*B p65, c‐Fos, c‐Jun, and NFAT1 were cloned into pcDNA3.1 vector. For dual‐luciferase assay, 3 × 10^5^ HEK 293T cells that seeded in 24‐well plates overnight were co‐transfected with 500 ng pGL3‐IL‐2 promoter, 10 ng pRL‐TK plasmid, and 500 ng TF expression plasmid (pcDNA3.1‐NF*κ*B p65, c‐Fos, c‐Jun or NFAT1) by Lipofectamine 2000 (Catalog #12 566 014, Invitrogen). After replacing the medium with fresh one at 5 h after transfection, cells were harvested and lysed by passive lysis buffer at 48 h after transfection, and the Luc activities were measured according to the manual of Dual‐Luciferase Reporter Assay System (Catalog #E1910, Promega).

### shRNA Interference, Lentivirus Packaging, and Cell Infection

The knockdown of endogenous human genes c‐Myc or GLS1 in Jurkat cells was achieved using microRNA‐based vector constructs with a GFP tag (GIPZ shRNAmiR lentivector system, GE healthcare). The guide strand sequences targeting c‐Myc and GLS1 were list in Table S3, Supporting Information, respectively. To construct a c‐Myc or GLS1 overexpression plasmid, the N‐terminal FLAG in duplicate tagged Nile tilapia c‐Myc or GLS1 gene sequences were codon optimized for mammalian cells and synthesized by GENEWIZ Co., Ltd. (Soochow, Jiangsu, China), and then fused following a truncated human low‐affinity nerve growth factor receptor used as a reporter molecule via a T2A peptide to facilitate the antibody staining of c‐Myc‐ or GLS1‐expressing cells. The DNA fragments were further cloned into the pLenti7.3 lentiviral vector (pLenti7.3/V5‐DEST Gateway Vector Kit, Thermo Fisher, Waltham, MA, USA), in which the human cytomegalovirus promoter was first replaced by the human elongation factor‐1*α* promoter. Constructs were verified by Sanger DNA sequencing (GENEWIZ). HEK293T/17 cells (ATCC) were co‐transfected with each of the lentiviral vector plasmids and the packaging plasmids using the calcium phosphate precipitation method. The supernatant was collected and then concentrated using ultracentrifugation at 25 000 rpm at 4 °C for 2 h (BECKMAN COULTER, Optima XPN‐100, Indianapolis, IN, USA). Jurkat cells were spin‐transduced with the lentiviral vector in culture medium with polybrene. At 4 h post transduction, the transduction medium was replaced with fresh complete medium and Jurkat cells were re‐suspended. The cells were harvested at designated time points for the various assays. Specifically, the cells were sorted using a fluorescence‐activated cell sorting system, and the sorted single‐cell clones were further expanded for use in vitro.

### Statistical Analysis

Prism version 8.0 software (GraphPad Software Inc., San Diego, CA, USA) was used for statistical analysis. The statistical significance of results was analyzed using two‐tailed, unpaired, Student's *t*‐test or two‐way analysis of variance analysis with significance indicated by *, *p* < 0.05; **, *p* < 0.01; ***, *p* < 0.001. Animal survival data were analyzed by log‐rank analysis.

## Conflict of Interest

The authors declare no conflict of interest.

## Author Contributions

K.L., X.J., W.D., J.L., W.L., and Y.Z. performed experiments and analyzed data. K.L., X.J., and W.D. wrote parts of the manuscript. X.W. and J.Y. gained the research funding, conceived the project, and designed experiments. J.Y. supervised the study and wrote the manuscript.

## Supporting information

Supporting Information

Supplemental Table 1

Supplemental Table 2

Supplemental Table 3

## Data Availability

The data that support the findings of this study are available in the supplementary material of this article.
