# Supplementary material for: Glutamine Metabolism Underlies the Functional Similarity of T Cells between Nile Tilapia and Tetrapod
Source: Adv Sci (Weinh). 2023 Mar 8;10(12):2201164. doi: 10.1002/advs.202201164 (PMC10131875; doi:10.1002/advs.202201164)
Supplement: Supplementary file 1 — Supporting Information [file ADVS-10-2201164-s001.pdf]

ADVANCED SCIENCE
Open Access

Supporting Information

for *Adv. Sci.*, DOI 10.1002/advs.202201164

Glutamine Metabolism Underlies the Functional Similarity of T Cells between Nile Tilapia and Tetrapod

*Kang Li, Xiumei Wei, Xinying Jiao, Wenhai Deng, Jiaqi Li, Wei Liang, Yu Zhang and Jialong Yang**

Supporting Information

Glutamine metabolism underlies the functional similarity of T cells between Nile tilapia and tetrapod

Kang Li [1, 2, #], Xiumei Wei [1, 2, #], Xinying Jiao [1, 2, #], Wenhai Deng [3, #], Jiaqi Li [1, 2], Wei Liang [1, 2], Yu Zhang [1], Jialong Yang [1, 2, *]

[#] K.L., X.W., X.J. and W.D. contributed equally to this work

[*] To whom correspondence should be addressed:

Jialong Yang, Ph.D., e-mail: jlyang@bio.ecnu.edu.cn

East China Normal University, Shanghai, 200241, China.

Phone: 86-021-34756904

This file contains 11 supplementary figures.

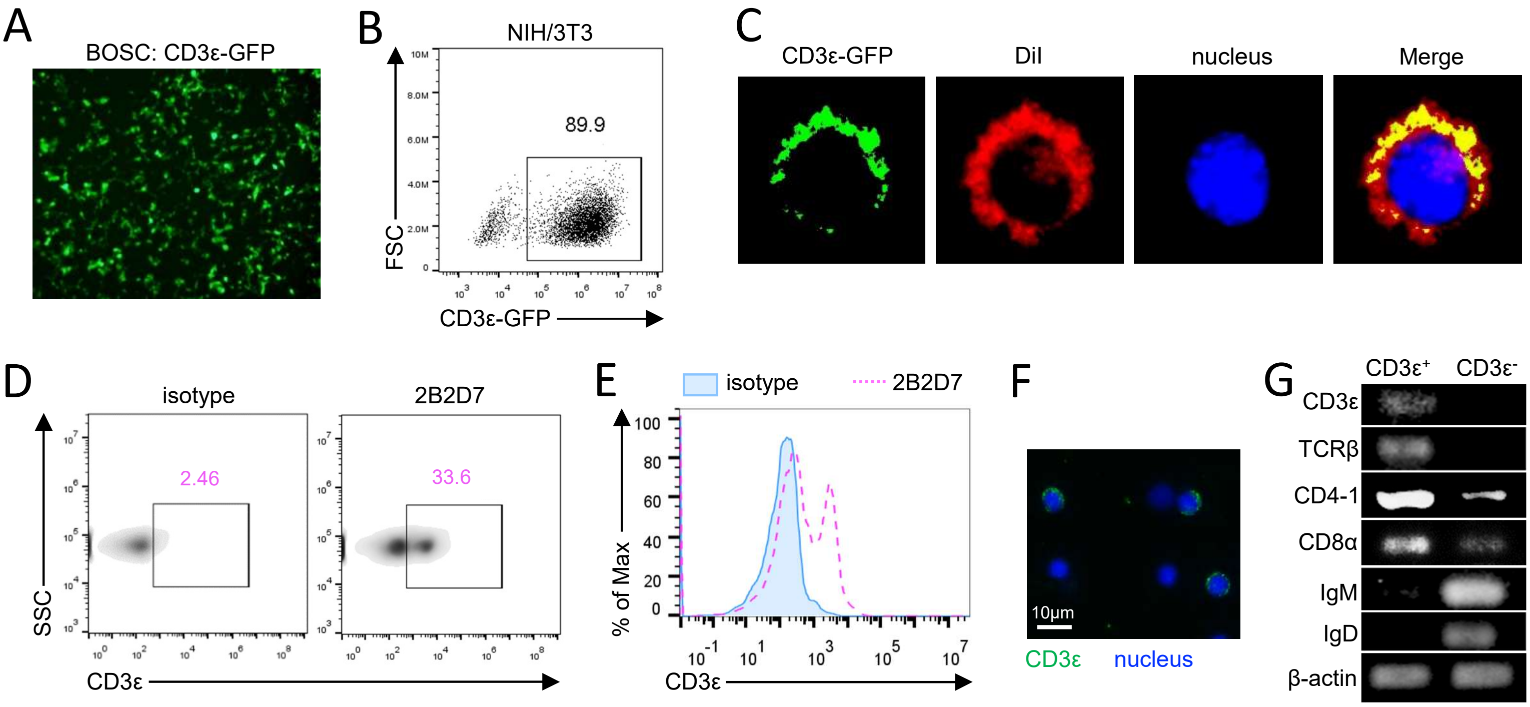

Figure S1. Development of mAb for tilapia CD3ε. **A**, BOSC23 cells were transfected to generate tilapia CD3ε retrovirus. **B**,**C**, NIH/3T3 cells infected with CD3ε retrovirus were analyzed by flow cytometry (**B**) or immunofluorescence (**C**). **D-G**, Splenocytes of mouse that immunized with tilapia CD3ε overexpressed-NIH/3T3 cells were fused with SP2/0 myeloma to develop CD3ε mAb. **D**,**E**, CD3ε^{+} cell percentage (**D**) and CD3ε expression (**E**) in tilapia spleen lymphocytes that stained with mAb 2B2D7. **F**, Immunofluorescence assay of tilapia spleen leukocytes using mAb 2B2D7. **G**, Expression of indicated genes in sorted CD3ε^{+} and CD3ε^{-} spleen lymphocytes.

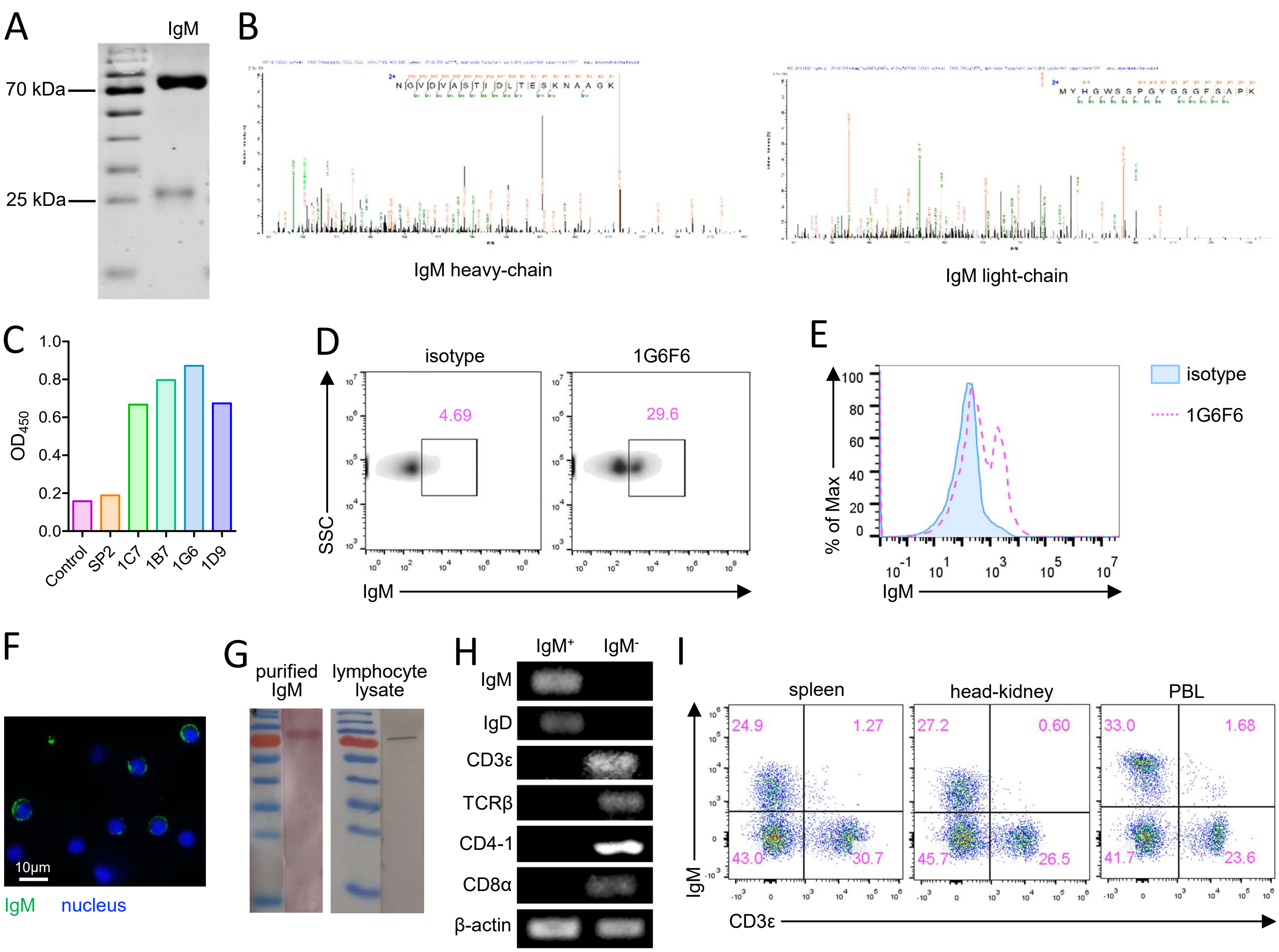

Figure S2. Development of mAb for tilapia IgM. A, SDS-PAGE shown the purified serum IgM of tilapia. **B**, The MALDI-TOF analysis of serum IgM heavy-chain and light-chain. **C-H**, Mouse splenocytes that immunized with purified tilapia IgM were fused with myeloma cells SP2/0, and tilapia IgM mAb was developed. **C**, Relative amount of anti-IgM mAbs in the supernatant of indicated SP2/0 or hybridomas detected by ELISA. **D**,**E**, Density plots (**D**) and overlaid histogram (**E**) stained with mAb 1G6F6 shown the IgM$^+$ cell percentage and IgM expression in tilapia spleen lymphocytes. **F**, Immunofluorescence staining shown the interaction of 1G6F6 with tilapia spleen leukocytes. **G**, Western blot shown the interaction of 1G6F6 with IgM heavy-chain in purified IgM or lymphocyte lysate. **H**, Expression profiles of indicated genes in sorted IgM$^+$ and IgM$^-$ spleen lymphocytes. **I**, Frequencies of T cells and IgM$^+$ B cells in indicated tissues of tilapia.

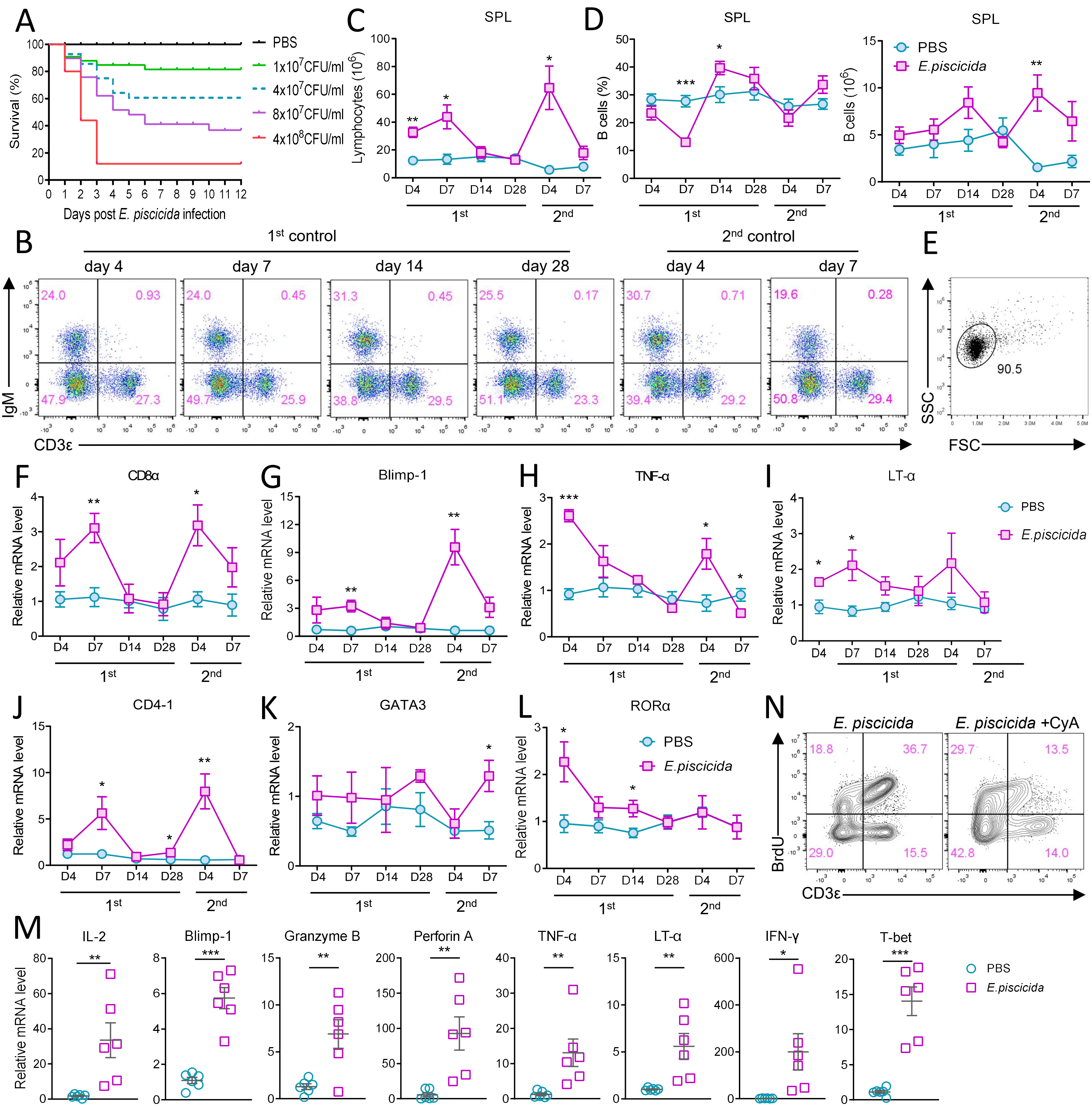

Figure S3. T cells play essential roles in anti-bacterial immune response of tilapia. **A**, Tilapia individuals were *i.p.* injected with different dose of *E.piscicida*. A Kaplan–Meyer survival plot shown the survival percentage of tilapia post-infection, n=23-25. **B-L**, Tilapia was *i.p.* injected with *E.piscicida* or not on day 0 and day 30, and spleen leukocytes were isolated at the indicated days for assay. **B**, Flow cytometry shown the frequency of T cells and IgM^+ B cells in the control tilapia. **C**, Absolute numbers of spleen lymphocytes, n=4-6. **D**, Frequencies (left) and absolute numbers (right) of IgM^+ B cells, n=4-6. **E**, Flow cytometry shown the purity of lymphocyte population in isolated spleen leukocyte. **F-L**, The relative mRNA levels of indicated molecules in spleen leukocytes, n=4-5. **M**, mRNA levels of indicated molecules in sorted spleen T cells from control or *E.piscicida*-infected tilapia on 5 DPI. **N**, *E.piscicida*-infected tilapia was *i.p.* injected with 10 mg/kg CyA on day 1, 2 and 3, and with 0.75 mg BrdU on day 5. BrdU and CD3ε staining in spleen lymphocytes was performed on 6 DPI. These experiments were repeated for three independent times. *: p<0.05, **: p<0.01, ***: p<0.001, determined by a two-tailed Student's t-test.

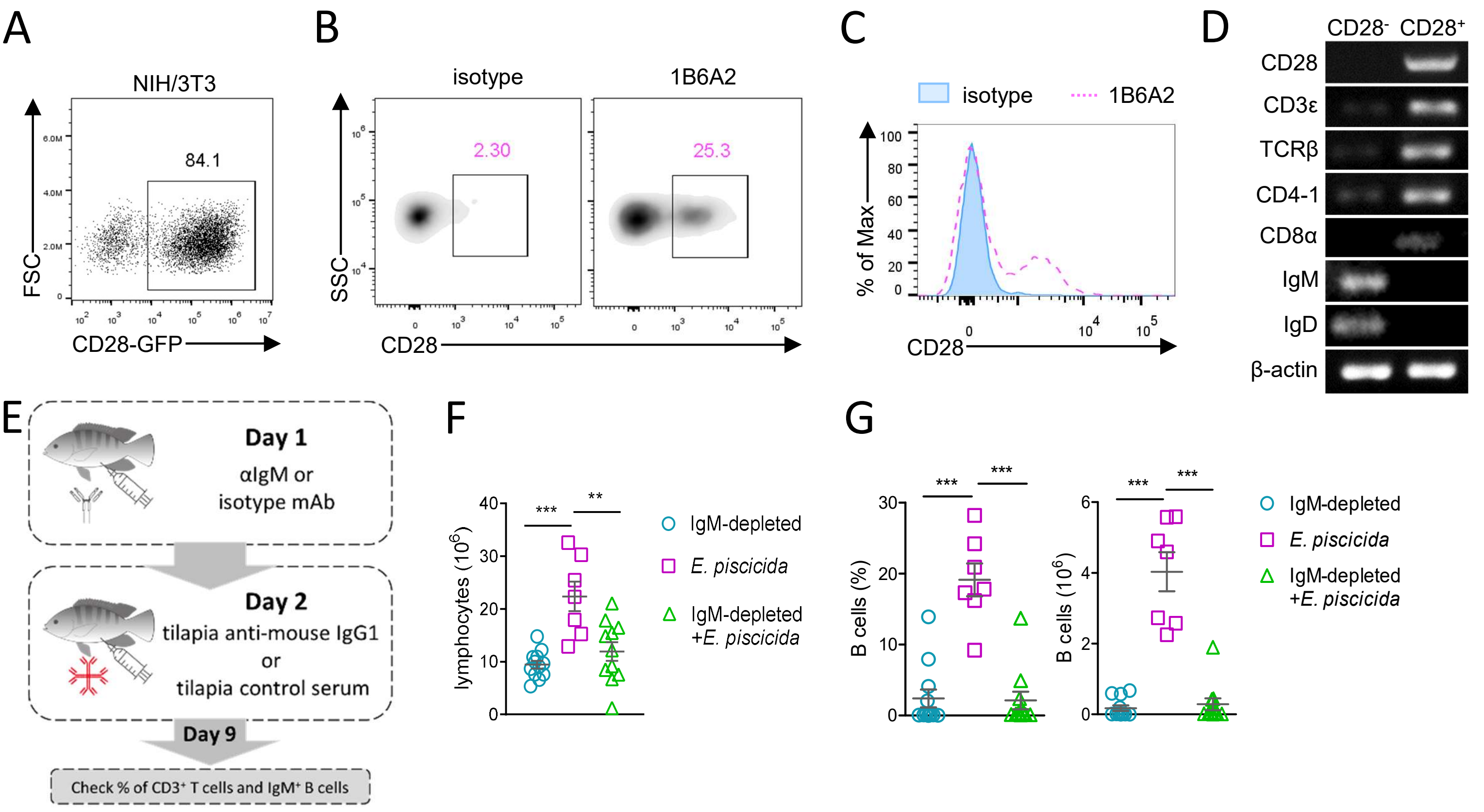

Figure S4. Development of tilapia CD28 mAb and IgM^{+} B-cell depletion. A, NIH/3T3 cells infected with tilapia-CD28 retrovirus were analyzed by flow cytometry. **B-D**, Splenocytes of mouse that immunized with tilapia CD28 overexpressed-NIH/3T3 cells were fused with SP2/0 myeloma to develop CD28 mAb. **B**,**C**, CD28^{+} cell percentage (**B**) and CD28 expression (**C**) in tilapia spleen lymphocytes that stained with mAb 1B6A2. **D**, Expression profiles of indicated genes in sorted CD28^{+} and CD28^{-} tilapia spleen lymphocytes. **E**, Strategy for IgM^{+} B cells depletion. **F**,**G**, IgM-depleted or non-depleted tilapia was infected with *E.piscicida* or not. Figures shown absolute numbers of spleen lymphocyte (**F**), and frequencies (left) and absolute numbers (right) of IgM^{+} B cells (**G**) in spleen on 7 DPI. These experiments were repeated for three independent times. **: p<0.01, ***: p<0.001, determined by a two-tailed Student's t-test.

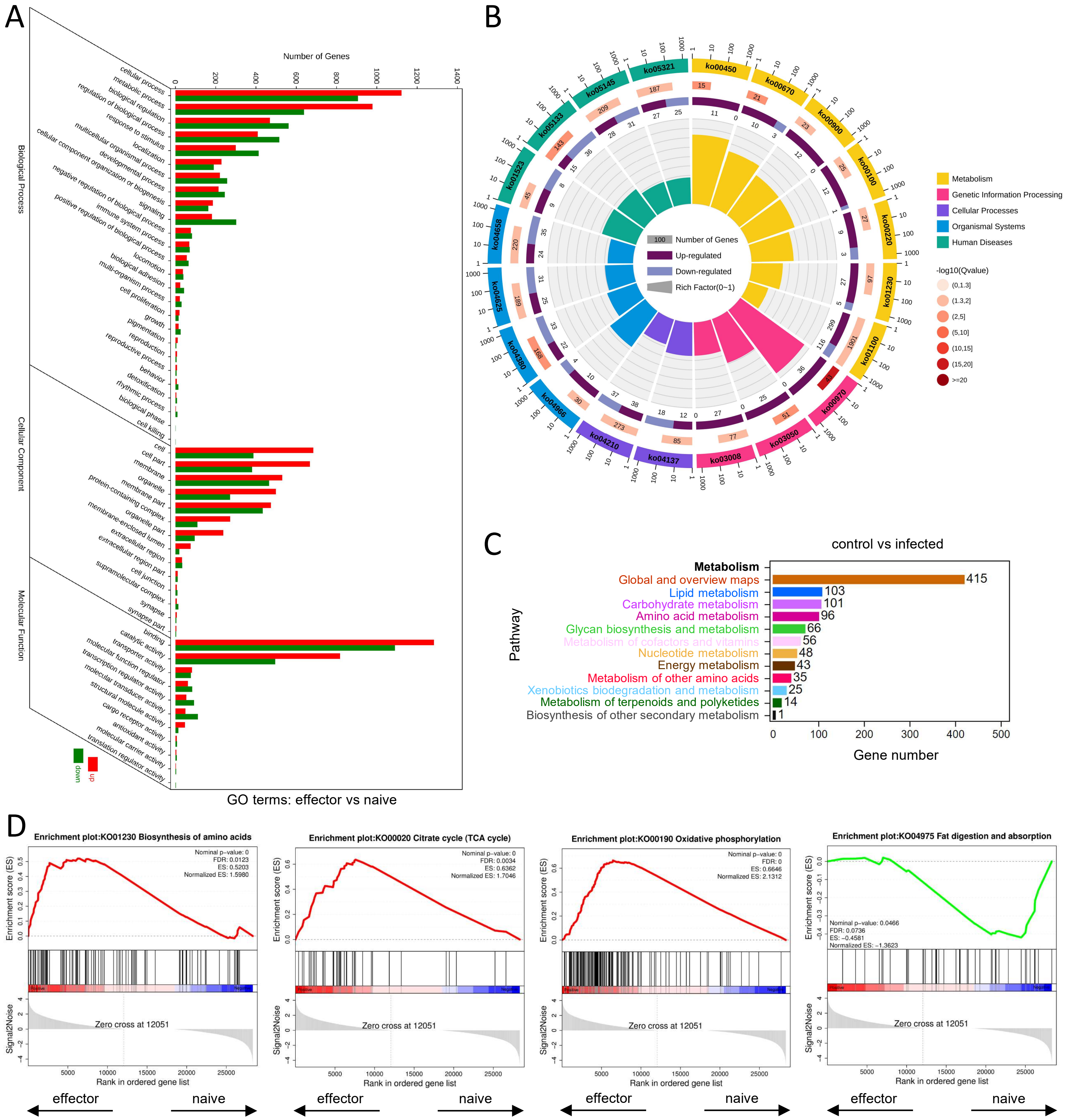

Figure S5. Transcriptional and metabolic profile of tilapia T cells. Spleen T cells were sorted from control or *E.piscicida*-infected tilapia on 5 DPI, and subjected for RNA-seq analyses. n=3. **A**, GO enrichment analysis of differentially expressed genes shown the processes involved. FDR＜0.05 and |log2FC|≥1. **B**, KEGG enrichment analysis of differentially expressed genes shown the Top-20 pathways involved. FDR<0.05 and |log2FC|>2. Scale bar represents z scores that were generated from −log10 (Qvalues). **C**, KEGG pathway enrichment analysis of differentially expressed genes shown the metabolic programs involved in the “Metabolic pathways” of **Fig 4C**. FDR<0.05 and |log2FC|>1. **D**, Gene-set enrichment analysis (GSEA) shown significant enrichment of gene sets associated with indicated metabolic programs.

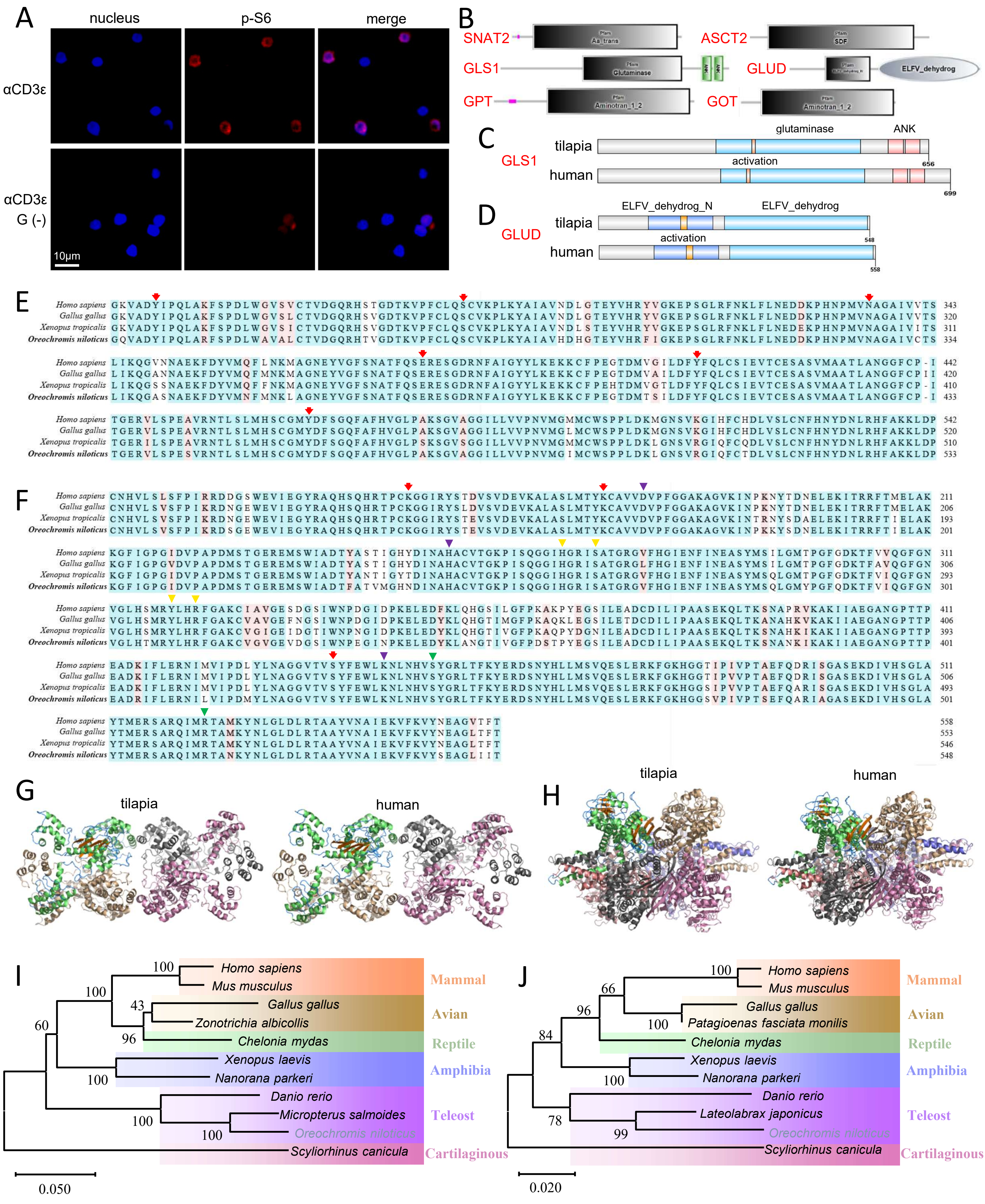
A
nucleus
p-S6
merge
αCD3ε
αCD3ε G (-)
10μm
B
SNAT2
Aa_trans
ASCT2
SDF
GLS1
Glutaminase
ANK
GLUD
ELFV_dehydrog
GPT
Aminotran_1_2
GOT
Aminotran_1_2
C
GLS1
tilapia
human
glutaminase
ANK
activation
656
699
D
GLUD
tilapia
human
ELFV_dehydrog_N
ELFV_dehydrog
activation
548
558
E
Homo sapiens
Gallus gallus
Xenopus tropicalis
Oreochromis niloticus
F
G
tilapia
human
H
tilapia
human
I
Homo sapiens
Mus musculus
Gallus gallus
Zonotrichia albicollis
Chelonia mydas
Xenopus laevis
Nanorana parkeri
Danio rerio
Micropterus salmoides
Oreochromis niloticus
Scyliorhinus canicula
Mammal
Avian
Reptile
Amphibia
Teleost
Cartilaginous
0.050
J
Homo sapiens
Mus musculus
Gallus gallus
Patagioenas fasciata monilis
Chelonia mydas
Xenopus laevis
Nanorana parkeri
Danio rerio
Lateolabrax japonicus
Oreochromis niloticus
Scyliorhinus canicula
Mammal
Avian
Reptile
Amphibia
Teleost
Cartilaginous
0.020

Figure S6. Tilapia possesses evolutionary conserved glutaminolysis pathway. A, Spleen leukocytes cultured in DMEM with or without glutamine were stimulated with 2 μg/mL CD3ε mAb. S6 phosphorylation was examined by immunofluorescence assay at 6 h. **B**, Domain prediction of glutaminolysis pathway components in tilapia. **C**,**D**, Domain organization of GLS1 (**C**) and GLUD (**D**) in tilapia and human. **E**, Multisequence alignment analysis of the functional domains from tilapia GLS1 with homologs in other vertebrates. Amino acid residues with 100% identity are in blue, and the similar amino acids are in red. The motifs that interact with glutamine are marked with red arrows. **F**, Multisequence alignment analysis of the functional domains from tilapia GLUD with homologs in other vertebrates. Amino acid residues with 100% identity are in blue, and the similar amino acids are in red. The motifs that interact with glutamine, NADH, ADP and GTP are marked with red arrows, purple triangles, green triangles and yellow triangles, respectively. **G**,**H**, Prediction of tertiary structures of GLS1 (**G**) and GLUD (**H**) from tilapia and human by SWISS-MODEL. **I**,**J**, Phylogenetic trees constructed with the amino acid sequences of GLS1 (**I**) and GLUD (**J**) from the indicated species. The trees were constructed using the neighbor-joining algorithm with the Mega program based on multiple sequence alignment by ClustalW. Bootstrap values of 1000 replicates (%) are indicated for the branches. The accession numbers of selected sequences are listed in Table S2. The experiment in **A** was repeated for three independent times.

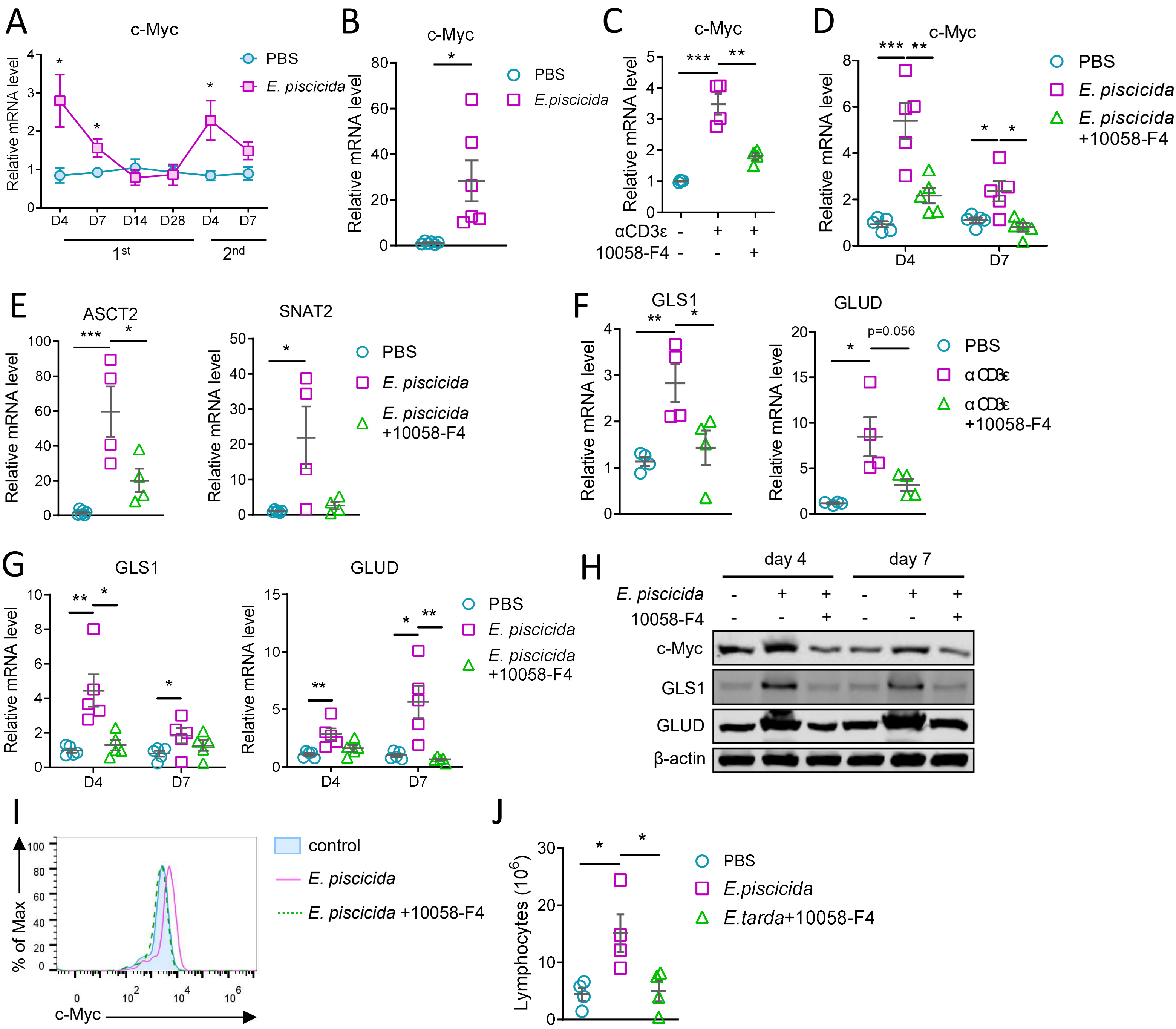

Figure S7. c-Myc is indispensable for glutaminolysis-controlled T-cell response of tilapia. A, Tilapia individuals were *i.p.* injected with *E.piscicida* on day 0 and 30, and mRNA levels of c-Myc in spleen leukocytes were examined, n=4-5. **B**, Relative mRNA level of c-Myc in T cells sorted from healthy or *E.piscicida*-infected tilapia on 5 DPI. **C**,**F**, Spleen leukocytes that stimulated with 2 μg/mL CD3ε mAb were treated with or without 5 nM 10058-F4 for 12 h, and relative mRNA levels of indicated molecules were examine. **D**,**E**,**G-J**, Healthy or *E.piscicida*-infected tilapia were *i.p.* injected with 10 mg/kg 10058-F4 on day 1, 3, 5 and 6, and spleen leukocytes were isolated for assay. **D**, Relative mRNA levels of c-Myc on indicated days. **E**, Relative mRNA levels of indicated molecules on 4 DPI. **G**, Relative mRNA levels of indicated molecules on 4 and 7 DPI. **H**, Protein levels of indicated molecules on 4 and 7 DPI. **I**, c-Myc expression in spleen T cells on 4 DPI. **J**, Absolute numbers of lymphocytes in spleen on 7 DPI. These experiments were repeated for three independent times. *: $p<0.05$, **: $p<0.01$, ***: $p<0.001$, determined by a two-tailed Student's t-test.

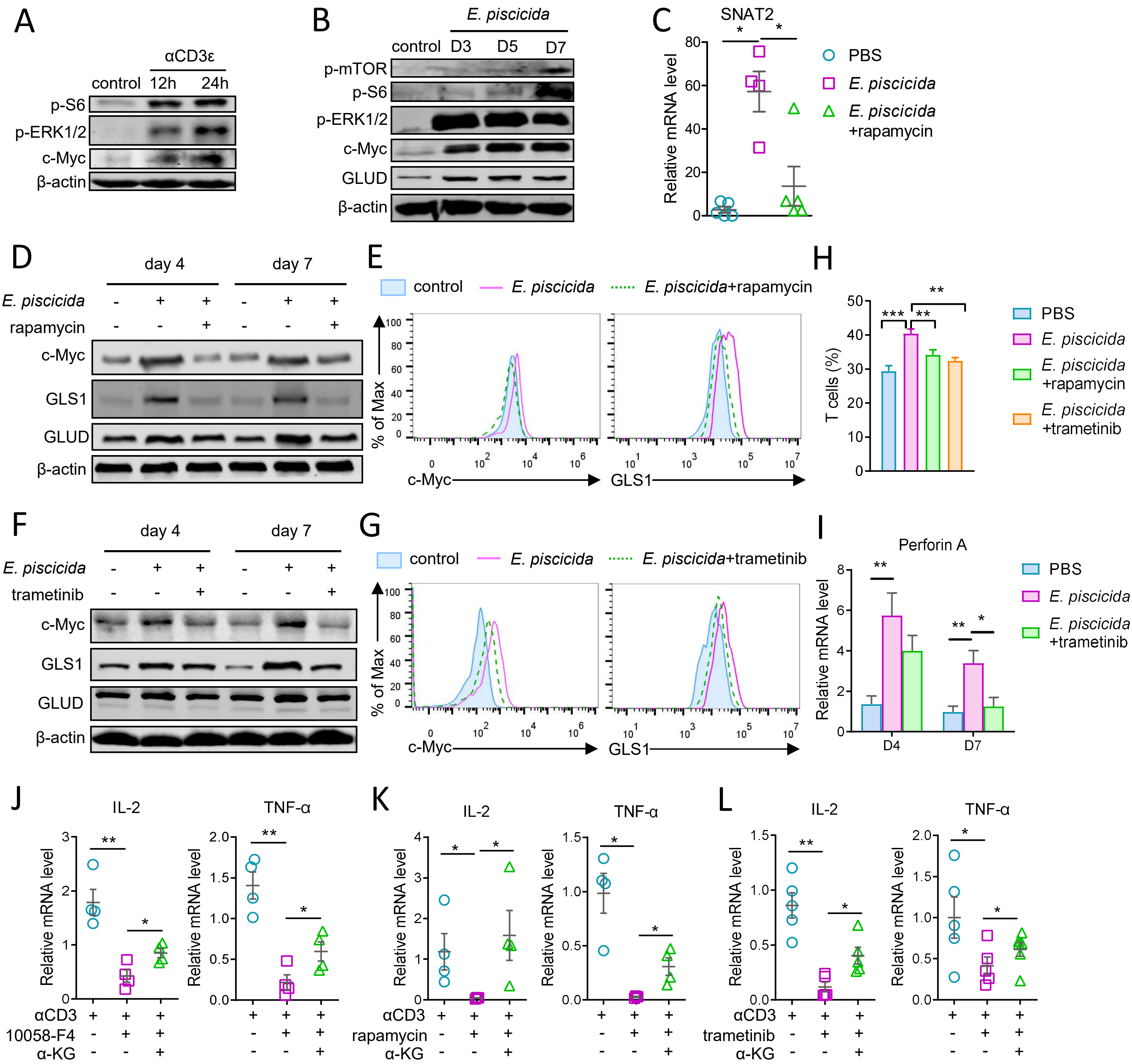

Figure S8. mTORC1 coordinates MAPK/ERK signal to promote c-Myc-regulated glutaminolysis and T-cell response. **A**,**B**, Protein levels of indicated molecules in 2 µg/mL CD3ε mAb-stimulated spleen leukocytes (**A**) or spleen leukocytes isolated from *E.piscicida*-infected tilapia (**B**). **C-I**, Healthy or *E.piscicida*-infected tilapia was *i.p.* injected with 1 mg/kg rapamycin or 0.1 mg/kg trametinib on day 1, 3, 5 and 6, and spleen leukocytes were isolated for assay. **C**, Relative mRNA levels of SNAT2 on 4 DPI. **D**,**F**, Protein levels of indicated molecules on 4 and 7 DPI. **E**,**G**, Expression of indicated molecules in spleen T cells on 4 DPI. **H**, Bar figure shown frequency of spleen T cells, n=4-9. **I**, Relative mRNA levels of Perforin A in spleen leukocytes on 4 DPI, n=5-6. **J-L**, Spleen leukocytes were stimulated with 2 µg/mL CD3ε mAb with or without 1mM α-KG, and 10058-F4 (**J**), rapamycin (**K**) or trametinib (**L**). Relative mRNA levels of indicated molecules were examined at 12 h after stimulation. These experiments were repeated for three independent times. *: p<0.05, determined by a two-tailed Student's t-test.

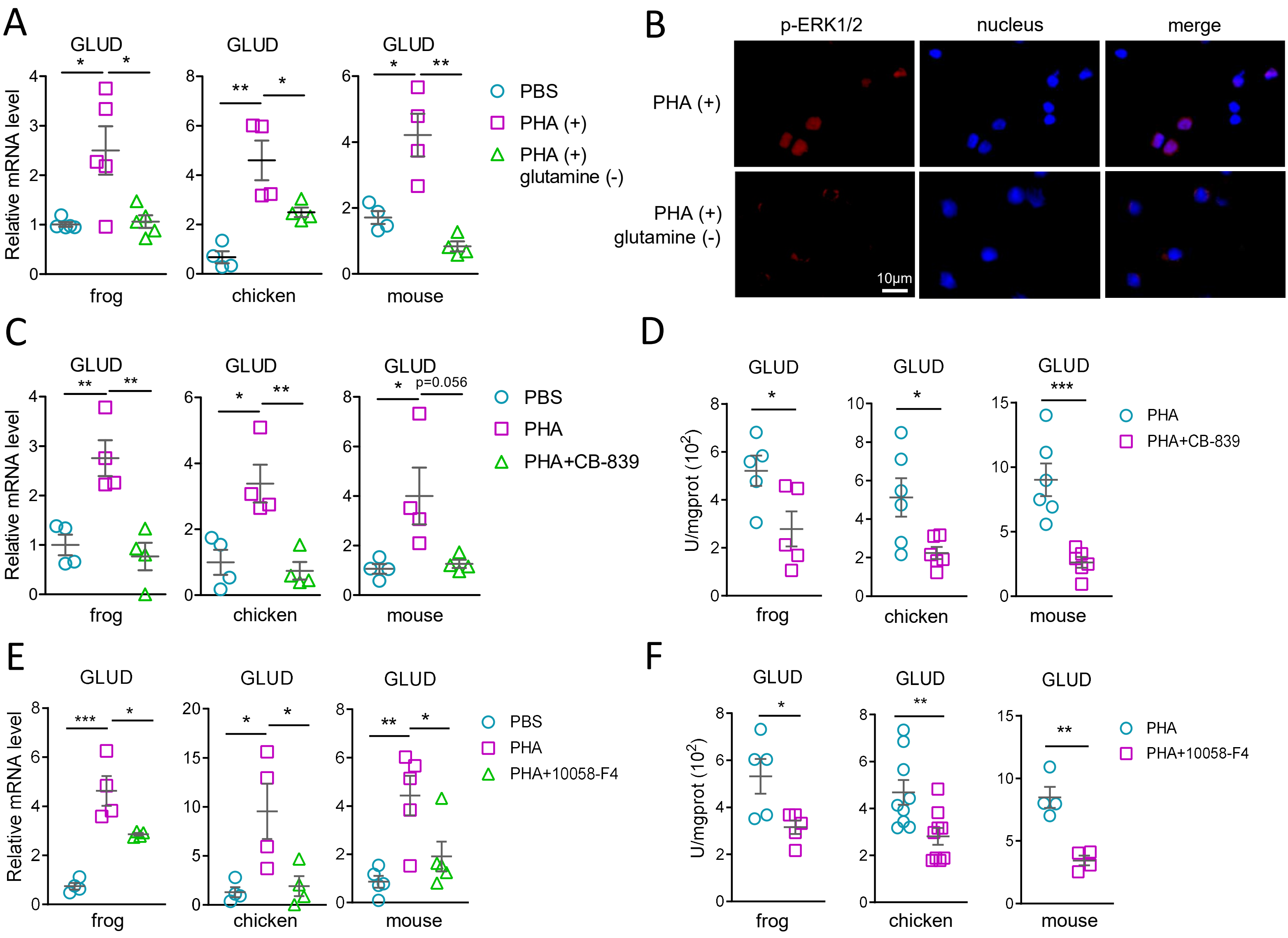

Figure S9. The dependency to glutaminolysis of T-cell response in different vertebrates. A,**B**, Spleen leukocytes of frog, chicken or mouse cultured in DMEM with or without glutamine were stimulated with 2 μg/mL PHA. **A**, The relative mRNA levels of GLUD at 12 h. **B**, Phosphorylation of ERK1/2 in mouse leukocytes was examined by immunofluorescence assay at 6 h post stimulation. **C-F**, Spleen leukocytes of frog, chicken or mouse were stimulated with 2 μg/mL PHA in the presence or absence of 10 nM CB-839 or 5 nM 10058-F4. The mRNA levels of GLUD at 12 h (**C**,**E**) and GLUD activity at 24 h (**D**,**F**) were examined. These experiments were repeated for three independent times. *: $p<0.05$, **: $p<0.01$, ***: $p<0.001$, determined by a two-tailed Student's t-test.

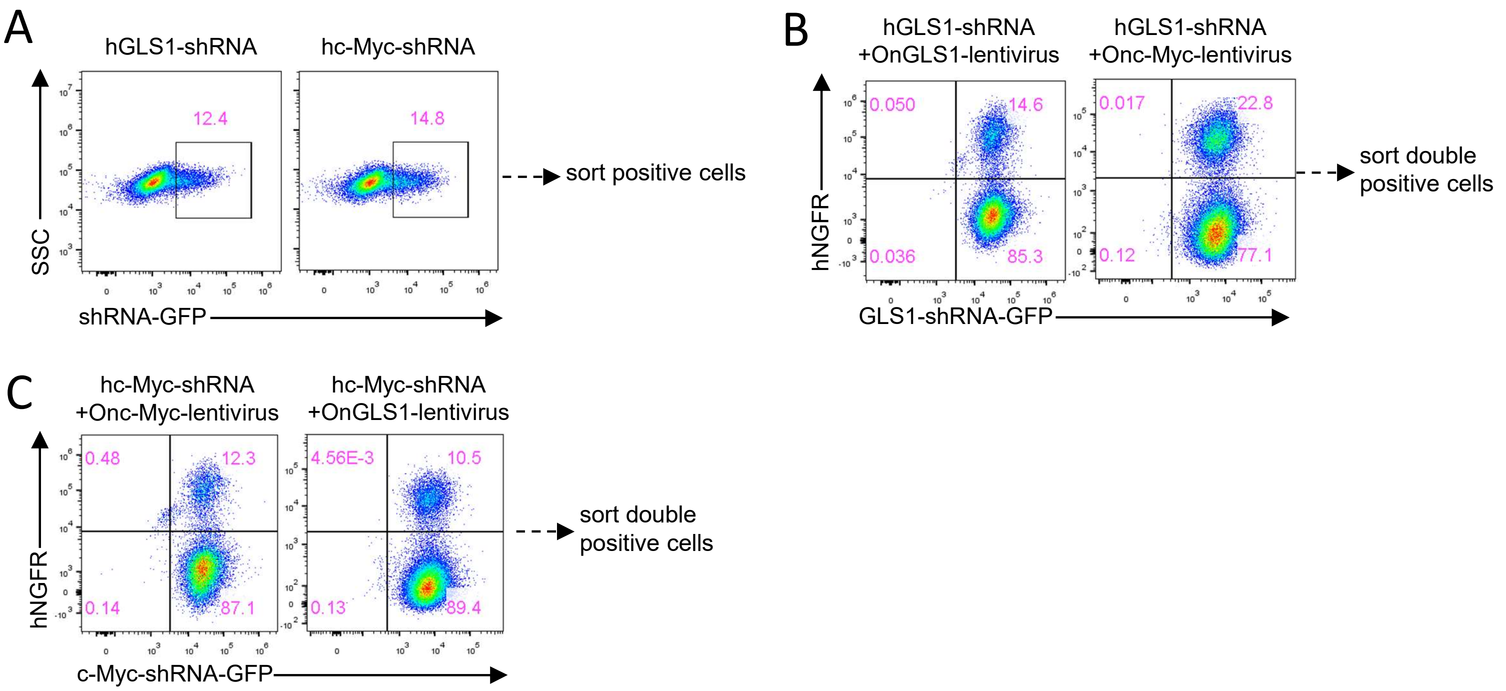

Figure S10. Construction of GLS1 or c-Myc knock down or overexpression Jurkat cells. **A**, Jurkat cells were transfected with GLS1-shRNA or c-Myc-shRNA plasmid, and GFP positive cells were sorted for expansion and analysis. **B**,**C**, GLS1 or c-Myc-interfered Jurkat cells were infected with lentivirus for tilapia GLS1 or c-Myc, and $GFP^{+}NGFR^{+}$ cells were sorted for expansion and analysis.

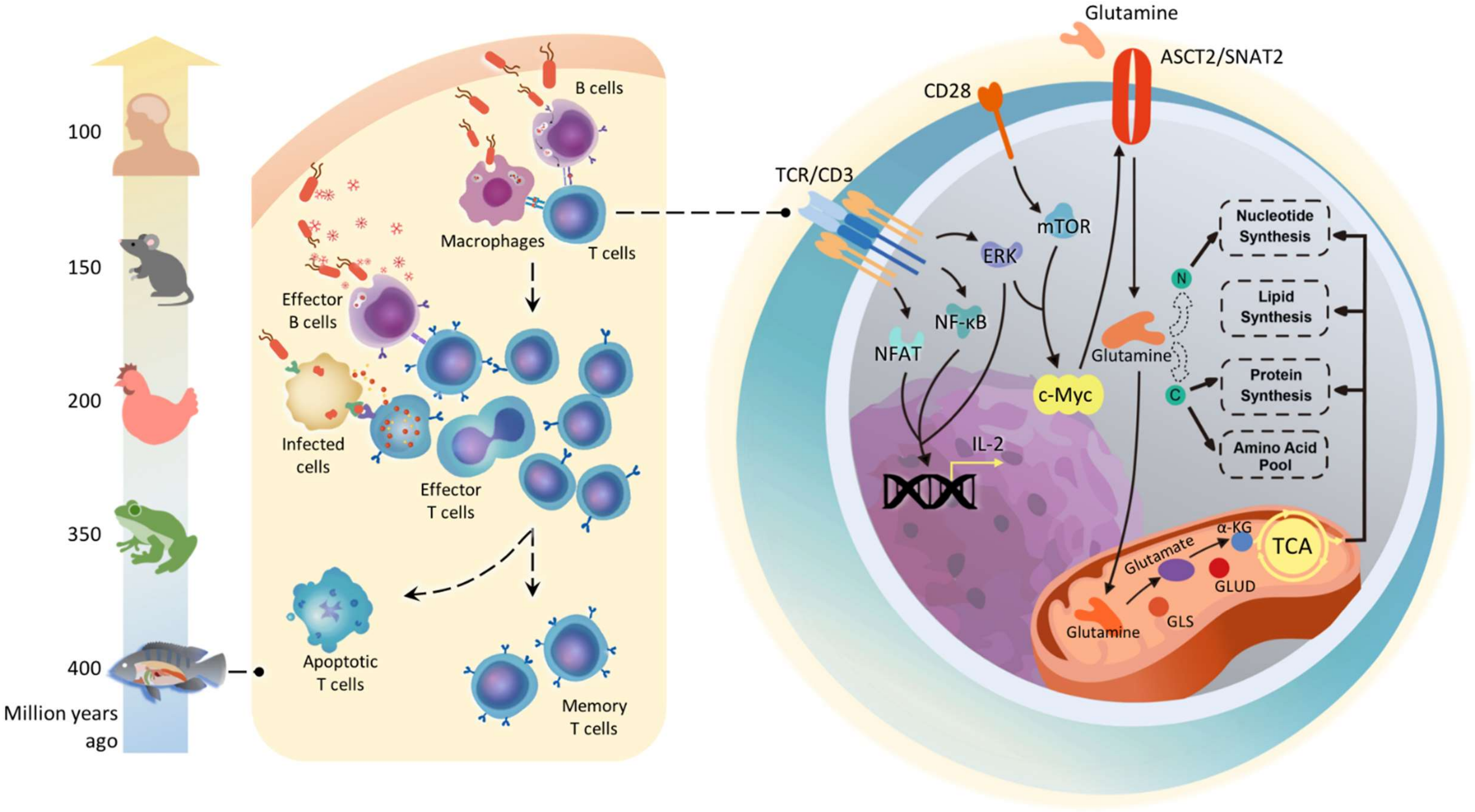

Figure S11. Glutamine metabolism underlies the functional similarity of T cells between Nile tilapia and tetrapod.
