# Supplementary material for: Glutamine Metabolism Underlies the Functional Similarity of T Cells between Nile Tilapia and Tetrapod
Source: Adv Sci (Weinh). 2023 Mar 8;10(12):2201164. doi: 10.1002/advs.202201164 (PMC10131875; doi:10.1002/advs.202201164)
Supplement: Supplementary file 3 — Supplemental Table 2 [file ADVS-10-2201164-s002.pdf]

ADVANCED SCIENCE
Open Access

Supporting Information

for *Adv. Sci.*, DOI 10.1002/advs.202201164

Glutamine Metabolism Underlies the Functional Similarity of T Cells between Nile Tilapia and Tetrapod

*Kang Li, Xiumei Wei, Xinying Jiao, Wenhai Deng, Jiaqi Li, Wei Liang, Yu Zhang and Jialong Yang**

Table S2. Information of genes used for sequence, phylogeny and structure analysis in present study

Source	Accession No.	Gene name	Species	Application
GenBank	XP_003455778.2	SNAT2	*Oreochromis niloticus*	DP
GenBank	XP_005455824.1	ASCT2	*Oreochromis niloticus*	DP
GenBank	XP_025758602.1	GLS1	*Oreochromis niloticus*	DP,DOA,MSA,3D,PA
GenBank	XP_003457513.1	GLUD	*Oreochromis niloticus*	DP,DOA,MSA,3D,PA
GenBank	XP_003438123.1	GPT	*Oreochromis niloticus*	DP
GenBank	XP_003442303.1	GOT	*Oreochromis niloticus*	DP
GenBank	AAG47842.1	GLS1	*Homo sapiens*	DOA,MSA,3D,PA
GenBank	AAA52525.1	GLUD	*Homo sapiens*	DOA,MSA,3D,PA
GenBank	XP_015143519.1	GLUD	*Gallus gallus*	MSA,PA
GenBank	NP_001011138.1	GLUD	*Xenopus tropicalis*	MSA
GenBank	XP_018081009.1	GLUD	*Xenopus laevis*	PA
GenBank	NP_001026419.1	GLS1	*Gallus gallus*	MSA,PA
GenBank	XP_031748808.1	GLS1	*Xenopus tropicalis*	MSA
GenBank	XP_018091724.1	GLS1	*Xenopus laevis*	PA
GenBank	AAH52724.1	GLUD	*Mus musculus*	PA
GenBank	OPJ84600.1	GLUD	*Patagioenas fasciata monilis*	PA
GenBank	XP_037760240.1	GLUD	*Chelonia mydas*	PA
GenBank	XP_018419338.1	GLUD	*Nanorana parkeri*	PA
GenBank	AAS92641.1	GLUD	*Danio rerio*	PA
GenBank	QOE77673.1	GLUD	*Lateolabrax japonicus*	PA
GenBank	XP_038638591.1	GLUD	*Scyliorhinus canicula*	PA
GenBank	NP_001074550.1	GLS1	*Mus musculus*	PA
GenBank	OPJ80170.1	GLS1	*Micropterus salmoides*	PA
GenBank	XP_037768471.1	GLS1	*Chelonia mydas*	PA
GenBank	XP_018426162.1	GLS1	*Nanorana parkeri*	PA
GenBank	NP_001038509.1	GLS1	*Danio rerio*	PA
GenBank	XP_005492821.2	GLS1	*Zonotrichia albicollis*	PA
GenBank	XP_038645067.1	GLS1	*Scyliorhinus canicula*	PA

Note:

MSA: multiple sequence alignment; PA: phylogenetic analysis; 3D: 3D structure analysis; DOA: domain organization analysis; DP: domain prediction.
