# Supplementary material for: Glutamine Metabolism Underlies the Functional Similarity of T Cells between Nile Tilapia and Tetrapod
Source: Adv Sci (Weinh). 2023 Mar 8;10(12):2201164. doi: 10.1002/advs.202201164 (PMC10131875; doi:10.1002/advs.202201164)
Supplement: Supplementary file 4 — Supplemental Table 3 [file ADVS-10-2201164-s004.pdf]

ADVANCED SCIENCE
Open Access

Supporting Information

for *Adv. Sci.*, DOI 10.1002/advs.202201164

# Glutamine Metabolism Underlies the Functional Similarity of T Cells between Nile Tilapia and Tetrapod

*Kang Li, Xiumei Wei, Xinying Jiao, Wenhai Deng, Jiaqi Li, Wei Liang, Yu Zhang and Jialong Yang**

Table S3. Information and sequence of the primers used in present study

| accession No. | Primer name | Forward (5'-3') | Reverse (5'-3') |
|---|---|---|---|
| KJ126772.1 | RT-On-β-actin | CGGAATCCACGAAACCACCTA | CCAGACGGAGTATTTACGCTCA |
| XM_003449297.5 | Rp-On-CD3ε | GAAGATCTGCCACCATGCTCAGCATGGGTGTC | CGGAATTCCTATCCCATCCTGTTGACC |
| KU884472.1 | Rp-On-CD28 | GAAGATCTGCCACCATGAGGATTTCATGGATGTTC | GGAATTCTTAGAAGTGTCTTTGTATATTCTGA |
| XM_003449297.5 | RT-On-CD3e | CTGGAGGACCAAAGTGACGCTG | CTCACACGCATTTCCTTCAACAT |
| AY428948.1 | RT-On-TNF-α | CGTCGTCGTGGCTCTTTGTT | TGGGGCTCTGTTTTGTCGC |
| NM_001287402.1 | RT-On-IFN-γ | GGGTGGTGTTTTGGAGTCGT | GTAGCGAGCCTGAGTTGTTGGTG |
| ENSONIT00000025699.1 | RT-On-CD8α | CATAACAGCAAAGGAAGGACAG | TACCTTGGATAAGTGACGCA |
| ENSONIT00000028270.2 | RT-On-IL-2 | ATGTCGAGACCCAGGGAAAC | CAGGCCACAGGTGACAGTTA |
| XM_005463226.4 | RT-On-Blimp-1 | ACTCTGCCGAAAGACGCC | TAACGCATCCAGTTGCTCCG |
| HM162889.1 | RT-On-TCRβ | TTCTACCCTGACCATGTCAAAA | TTCCCATTGTTTGGCATAGAC |
| ENSONIT00000016394 | RT-On-CD4-1 | CCAAGGGAAACAGAGAAGGAAA | AAGGGATGGTGAGAGGTGAAAC |
| KC677037.1 | RT-On-IgM | TGGCTTGTGGATGACGAGGA | AGCACTTGGAGTCTTGGTTGATG |
| KF530821.1 | RT-On-IgD | CTTGCTTGAGGGCTCTAATGAAC | CAGGTAAAGGTGGCTCTGGACA |
| KU884472.1 | RT-On-CD28 | AATGGGATGCTGTGGAACTGC | ACTCCTGTGGGATTGCGTGTG |
| XM_003448658.4 | RT-On-T-bet | ACCTCGGTCACCCAACTAAATC | CACCCACACCTCCCTCAAAT |
| XM_005464289.3 | RT-On-LT-α | GGTGCCCAGAGATGGCTTGTA | TTGTGTGGATTGATGAGAGGAGAGT |
| XM_025906636.1 | RT-On-Perforin A | TTCTCCGACAGGAAGACCAAG | TGTAGTCACTGATGGCGTCTC |
| XM_003448073.5 | RT-On-GATA3 | AACAGCATCCTGGCTCACAT | GCTCGGACAGAGTTTCCATAGT |
| XM_003440430.5 | RT-On-RORα | TGTAAGGGCTTTTTCAGGAGGA | GTTGCGGCTTGTGCGGT |
| XM_003439678.4 | RT-On-Granzyme B | GAGCATTTTGTGGTGACTGCTGC | CACGGTCTGCGAGAGGAATAGGT |
| XM_005455767.4 | RT-On-ASCT2 | TAGCCACAGCCTTTGGAACA | CTGATTTGCTTGGATACGCC |
| XM_003455730.5 | RT-On-SNAT2 | TGAACACACGGGCAAGAAGAA | AGGAGAATCACAAACAGGGCG GGATCG |
| XM_003438803.4 | RT-On-GLS1 | GCACGAGTTTGGCAGTGAGCA | ATGGGGTTGTGTGGTTTATCTTCC |
| XM_005448983.3 | RT-On-c-Myc | CTCGGTGGTTTTCCCGTATCC | CGTGTCCAGTCCCAAATCCTT |
| XM_003457465.4 | RT-On-GLUD | CTTCGTCATCCAGGGTTTCG | GCCAGTTTGTAGTCCTCCAGC |
| XM_025905613.1 | R-On-p65 | CGGGATCCATGGACCCTGGATATGGATGGA | CCAAGCTTAGTCGGGTGTCCTGACACAAA |

| | | | |
|---|---|---|---|
| XM_005476930.3 | R-On-c-Fos | CGGAATTCTATGTATCAAAACAACCTG | CGGGATCCTAAGGCTAGGAGAGTCG |
| XM_005458069.4 | R-On-Jun | CCGGAATTCATGTCCAGAAAAATGGAAGC | CGCGGATCCGTAAGCTTGGAGCTGCTGC |
| XM_013276256.3 | R-On-NFAT | CCGGAATTCATGACCTCCTTTTACGACGAAA | CCCAAGCTTTGTCTGACCTCTGGCCTGAAC |
| NM_001088953.2 | RT-Xl-β-actin | ATGCTCCCCGTGCTGTTTT | CTCGCAGTTCATTGTAGAAGGTGT |
| EF513165.1 | RT-Xl-IL-2 | GGCAAACAAGCCAACAACACT | AAGCAGCAAAGGGCACTTATTC |
| XM_018251706.1 | RT-Xl-IFN-γ | TCTGTTTCTGTGTCATTCTTTATTGG | TTTTTCCTCTCCTTCCTCTTTCC |
| NM_001093554.1 | RT-Xl-GLUD | CTGATAGTGTCTTGCCGCTTTC | TCTTCTGCTCCTCCGTCTCG |
| NM_205518.2 | RT-Gg-β-actin | TGAACCCCAAAGCCAACAGA | CAGGACTCCATACCCAAGAAAGAT |
| NM_204153.1 | RT-Gg-IL-2 | TGATGGCGTGAAGAAGGTG | CTGGGTCTCAGTTGGTGTGTAG |
| NM_205149.1 | RT-Gg-IFN-γ | ATCATACTGAGCCAGATTGTTTCG | TCAAGTCGTTCATCGGGAGC |
| XM_015288033.2 | RT-Gg-GLUD | GCAACTGGTCGTGGTCTCTTCC | CTGTTTCCAAAATACTGCCCTCAA |
| BC138614.1 | RT-Mm-β-actin | TCGTGCGTGACATCAAAGAGAA | CCAAGAAGGAAGGCTGGAAAA |
| NM_008366.3 | RT-Mm-IL-2 | TCTACAGCGGAAGCACAGCA | CCTCAGAAAGTCCACCACAGTTG |
| BC119063.1 | RT-Mm-IFN-γ | TAACTCAAGTGGCATAGATGTGGA | TCAGCAGCGACTCCTTTTCC |
| BC057347.1 | RT-Mm-GLUD | TTACACGGAGGTTCACTATGGAGC | CAAAGCCTGGTGTCATTCCTAAAA |
| NM_002467.6 | Si-Ju-Myc | UUUGCUCCUCUGCUUGGACGGA | |
| BC038507.1 | Si-Ju-GLS1 | AUUUACCAUAGGAUUAUGUGGU | |

Note:

RT: qPCR; Rp: Retrovirus packaging;R: Recombinant; Si: shRNA interference; On: *Oreochromis niloticus*; Xl: *Xenopus laevis*; Gg: *Gallus gallus*; Mm: *Mus musculus*; Ju: Jurkat.
